# Elevated CO_2_ and warming intensify plant reliance on soil nitrogen reserves despite intensive fertilization

**DOI:** 10.1038/s41467-026-75147-w

**Published:** 2026-07-08

**Authors:** Yuhao Zhu, Li Wan, Longlong Xia, Klaus Butterbach-Bahl, Michael Dannenmann, Clemens Scheer, Nadine K. Ruehr, Benjamin Wolf, Xiaoyuan Yan, Zhijun Wei, Peter B. Reich, Yiqi Luo, Pete Smith, Josep Peñuelas, Michael Schloter, Stefanie Schulz, Ralf Kiese

**Affiliations:** 1 Institute for Meteorology and Climate Research (IMK-IFU), Karlsruhe Institute of Technology, Garmisch-Partenkirchen, Germany; 2 Key Laboratory of Mountain Surface Processes and Ecological Regulation, Institute of Mountain Hazards and Environment, Chinese Academy of Sciences, Chengdu, China; 3 Key Laboratory of Acidified Soil Amelioration and Utilization, Ministry of Agriculture and Rural Affairs; Institute of Soil & Fertilizer and Resources & Environment, Jiangxi Academy of Agricultural Sciences, Nanchang, China; 4 State Key Laboratory of Soil and Sustainable Agriculture, Changshu National Agro-Ecosystem Observation and Research Station, Institute of Soil Science, Chinese Academy of Sciences, Nanjing, China; 5 Pioneer Center Land-CRAFT, Department of Agroecology, Aarhus University, Aarhus, Denmark; 6 Department of Forest Resources, University of Minnesota, St. Paul, MN USA; 7 Institute for Global Change Biology, University of Michigan, Ann Arbor, MI USA; 8 Soil and Crop Sciences Section, School of Integrative Plant Science, Cornell University, Ithaca, NY USA; 9 Institute of Biological and Environmental Sciences, University of Aberdeen, Cruickshank Building, St Macha r Drive, Aberdeen, UK; 10 CSIC, Global Ecology Unit CREAF-CSIC-UAB, Barcelona, Spain; 11 Helmholtz Center Munich, Research Unit Comparative Microbiome Analysis, Neuherberg, Germany

**Keywords:** Environmental impact, Element cycles

## Abstract

Understanding how ecosystems sustain plant nitrogen (N) supply under climate change is critical, yet whether increasing plant N demand is met by external inputs or mobilization of soil N reserves remains unresolved. Here we show that climate change increases plant reliance on soil N reserves despite intensive fertilization. Using a two-year ^15^N-tracing experiment combining elevated CO_2_, warming, and drought in a montane grassland, we found that plants obtained 82–88% of their N from soil and acquired 4.6–7.3 times more N from soil than from fertilizer despite high N inputs. Elevated CO_2_ and warming increased plant uptake of soil-derived N but not fertilizer N. Consequently, plant N export exceeded fertilizer inputs, causing ecosystem N deficits and depletion of soil N stocks, with the strongest soil N mining under combined elevated CO_2_ and warming. Our findings reveal that climate change accelerates biological mining of soil N reserves, potentially constraining the long-term sustainability of intensively managed agroecosystems.

## Abstract (web summary)

A field experiment shows that climate change increases plant reliance on soil nitrogen reserves despite intensive fertilization, accelerating depletion of soil nitrogen stocks and potentially threatening the long-term sustainability of managed ecosystems.

## Introduction

Terrestrial ecosystems are increasingly exposed to concurrent global change drivers, with elevated atmospheric CO_2_ (eCO_2_), rising temperatures (warming), and altered precipitation regimes—particularly drought—emerging as dominant components of anthropogenic climate change^1–3^. Understanding how ecosystems sustain plant nitrogen (N) supply under these conditions is essential for predicting future productivity. However, it remains unclear whether climate-driven changes in plant N demand are met primarily by external inputs or by the mobilization of soil organic nitrogen (SON) reserves (i.e., biological soil N mining). Because N availability constrains productivity across most terrestrial ecosystems^4,5^, the balance between plant N demand and ecosystem N supply governs biosphere responses to global change^6,7^. Climate drivers, such as eCO_2_ and warming, are expected to stimulate plant growth and increase N demand^8–10^, but the sources from which this demand is satisfied remain poorly resolved. This distinction is crucial, as SON pools are often orders of magnitude larger than annual external inputs^11^, and shifts in ecosystem N sourcing could fundamentally alter soil nutrient stocks and long-term ecosystem sustainability.

Prevailing frameworks assume that external N inputs buffer ecosystems against N limitation under climate change^5,12^. In managed ecosystems, fertilizer application is expected to sustain productivity by directly supplying plant-available N, thereby meeting increased plant demand under eCO_2_ and warming. Experimental and synthesis studies have shown that N addition can enhance both plant N concentration and uptake under eCO_2_, warming, and drought^12–14^. At the same time, eCO_2_ and warming can stimulate microbial activity and accelerate soil organic matter decomposition, increasing N mineralization and the availability of soil-derived N to plant uptake^15,16^. Although drought generally suppresses microbial processes, subsequent rewetting events can trigger pulses of SON mineralization^13^. These responses point to an alternative pathway in which increased plant demand under climate change may be met not by external inputs, but by enhanced mobilization of internal SON reserves^17^. However, no previous study has simultaneously quantified the relative contributions of fertilizer-derived versus soil-derived N to plant uptake under eCO_2_, warming and drought within a single experimental framework, contributing to major uncertainties in projections of nitrogen-climate feedbacks in terrestrial ecosystems.

A shift toward greater reliance on soil-derived N would represent a fundamental reorganization of ecosystem N sourcing, whereby increased plant demand is met through accelerated mobilization of SON reserves rather than external inputs. This process, conceptualized as biological mining of soil N^5^, may sustain productivity in the short term but risks depleting long-term soil N stocks and creating persistent ecosystem N deficits^18,19^. Emerging evidence suggests that climate change may increase the contribution of soil-derived N to plant N pools^20^. Experimental translocation of intact plant–soil cores to warmer and drier environments has demonstrated substantial increases in gross N mineralization in montane grasslands, accompanied by enhanced plant N uptake^21^. In addition, warming can increase fertilizer N losses via denitrification, potentially amplifying the relative importance of SON-derived N for plant uptake^21–23^. Nevertheless, direct evidence is lacking on quantifying whether climate change shifts plant N acquisition toward fertilizer-derived or soil-derived sources, particularly under combined drivers such as eCO_2_, warming, and drought.

Montane grasslands in the pre-Alpine region provide an ideal system to address this question. These ecosystems contain large SON stocks (1.2–1.6 kg N m⁻^2^), reflecting long-term organic fertilization and slow decomposition under cool and moist conditions^20^. They sustain high productivity and are increasingly exposed to warming, elevated CO_2_, and more frequent droughts^11^. Despite substantial slurry N inputs (~180–200 kg N ha⁻^1^ yr⁻^1^), N use efficiency is low, with 40–50% of applied N lost primarily through gaseous emissions^17^, suggesting that SON reserves may contribute substantially to plant nutrition. In addition, slurry applications simultaneously introduce both carbon and nitrogen into soils, potentially stimulating microbial activity and altering soil N transformation under climate change. Previous work indicates that warming can accelerate N turnover in these systems^21^, yet plant N responses to eCO_2_ and interacting climate drivers remain poorly constrained. Moreover, the interactive effects of eCO_2_, warming, and drought on soil–plant N dynamics, whether additive, antagonistic, or synergistic, remain unclear. Closing these knowledge gaps is critical for developing targeted management strategies that enhance the resilience of montane grasslands to concurrent climate pressures while safeguarding soil fertility and ecosystem productivity.

Here, we evaluate how climate change alters ecosystem N sourcing using a two-year multifactor experiment that combines eCO_2_, warming, and drought with in-situ ^15^N tracing in intact plant–soil mesocosms from a montane grassland in the southern German Alps (Supplementary Fig. 1). Contrary to prevailing expectations, we showed that climate change consistently increased plant reliance on soil-derived N despite intensive fertilizer inputs. Across all treatments, plants obtained the majority of their N from soil, acquiring substantially more N from SON than from fertilizer even under high N inputs. Warming and eCO_2_ increased plant uptake of soil-derived N while leaving fertilizer-derived N acquisition largely unchanged. Consequently, plant N export exceeded fertilizer inputs and generated sustained negative ecosystem N balances and progressive depletion of soil N stocks. Multifactor climate treatments amplified this response, with the strongest soil N mining occurring under combined warming and eCO_2_. This mechanism challenges the prevailing assumption that external N inputs can buffer ecosystems against increasing N demand. Accounting for climate-driven shifts in ecosystem N sourcing will be essential for improving predictions of nutrient–nitrogen feedbacks and for developing sustainable N management strategies under ongoing global change.

## Results

### Divergent responses of plant biomass and N concentration to climate change

**Fig. 1:**
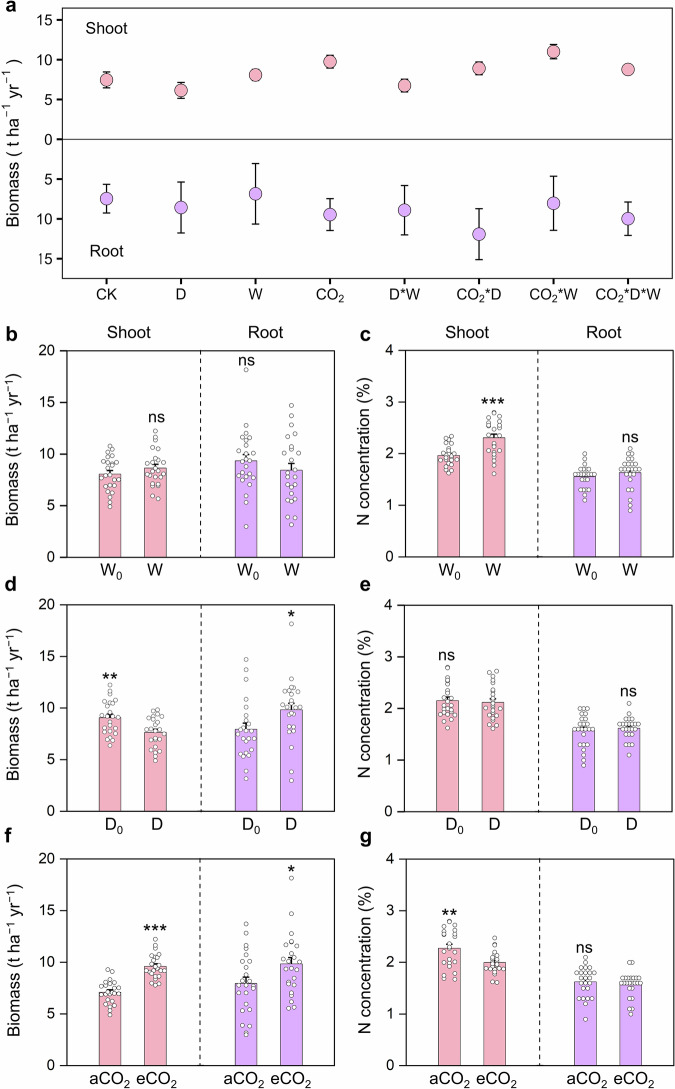
Grass shoot and root biomass (a, b, d, f) and their N concentration (c, e, g) in different climate change manipulation treatments. **a** Values are two-year averaged from 2020 to 2022. W_0_, ambient temperature; W, warming; D_0_, ambient rainfall; D, drought; aCO_2_, ambient CO_2_ concentration; CO_2_ (a) and eCO_2_ (f, g), elevated CO_2_ concentration. In **b**−**g**, values are recalculated across all levels of the other treatments to assess the effects of single climate change factor. Bars show means ± SE **a**−**g**. Significant differences between groups were determined using one‑way ANOVA followed by a two‑sided Tukey HSD test (*0.01 ≤ *P* < 0.05, **0.001 ≤ *P* < 0.01, ***P < 0.001, ^ns^0.05 ≤ *P*). The exact *P* values of statistical tests are provided in the Source Data file. In a, *n* = 6 (biological replicates), and in b − g, *n* = 24 (biological replicates show*n* by dots). Source data are provided as a Source Data file.

Across the study period (2020.07–2022.07), two-year mean shoot and root biomass ranged from 7.5 to 11 t ha⁻^1^ yr⁻^1^ and 6.9 to 12 t ha⁻^1^ yr⁻^1^ among treatments, respectively (Fig. 1a, Supplementary Tables 2–3). Climate drivers induced contrasting responses in above- and belowground biomass. Drought significantly reduced shoot biomass by 15.8% (*P* < 0.01), while increasing root biomass by 23.8% (*P* < 0.05; Fig. 1d, Supplementary Tables 4–5), suggesting a shift in biomass allocation under water stress. In contrast, warming produced opposite effects, modestly increasing shoot biomass by 7.3% while reducing root biomass by 9.7%, although these changes were not statistically significant (Fig. 1b). eCO_2_ significantly enhanced both shoot and root biomass by 24–35.2% (*P* < 0.05; Fig. 1f), exceeding the effects of warming or drought applied individually. Consequently, under combined treatments, eCO_2_ largely offset the negative effects of drought and warming, resulting in net increases in plant biomass (Supplementary Fig. 2; Supplementary Table 6). Interactive effects of climate drivers on biomass were predominantly additive (Supplementary Table 4 and 6).

In contrast to biomass responses, plant N concentrations exhibited distinct patterns. eCO_2_ significantly reduced shoot N concentration (*P* < 0.001; Fig. 1g), which was increased by warming (*P* < 0.001; Fig. 1c). Root N concentration remained largely unchanged across all treatments despite substantial variation in root biomass (Fig. 1e). Likewise, combinations of climate drivers did not significantly affect N concentrations in either shoots or roots (Supplementary Fig. 2), indicating different responses between biomass production and tissue N concentration under climate change.

### Climate change intensifies plant reliance on soil-derived nitrogen

**Fig. 2:**
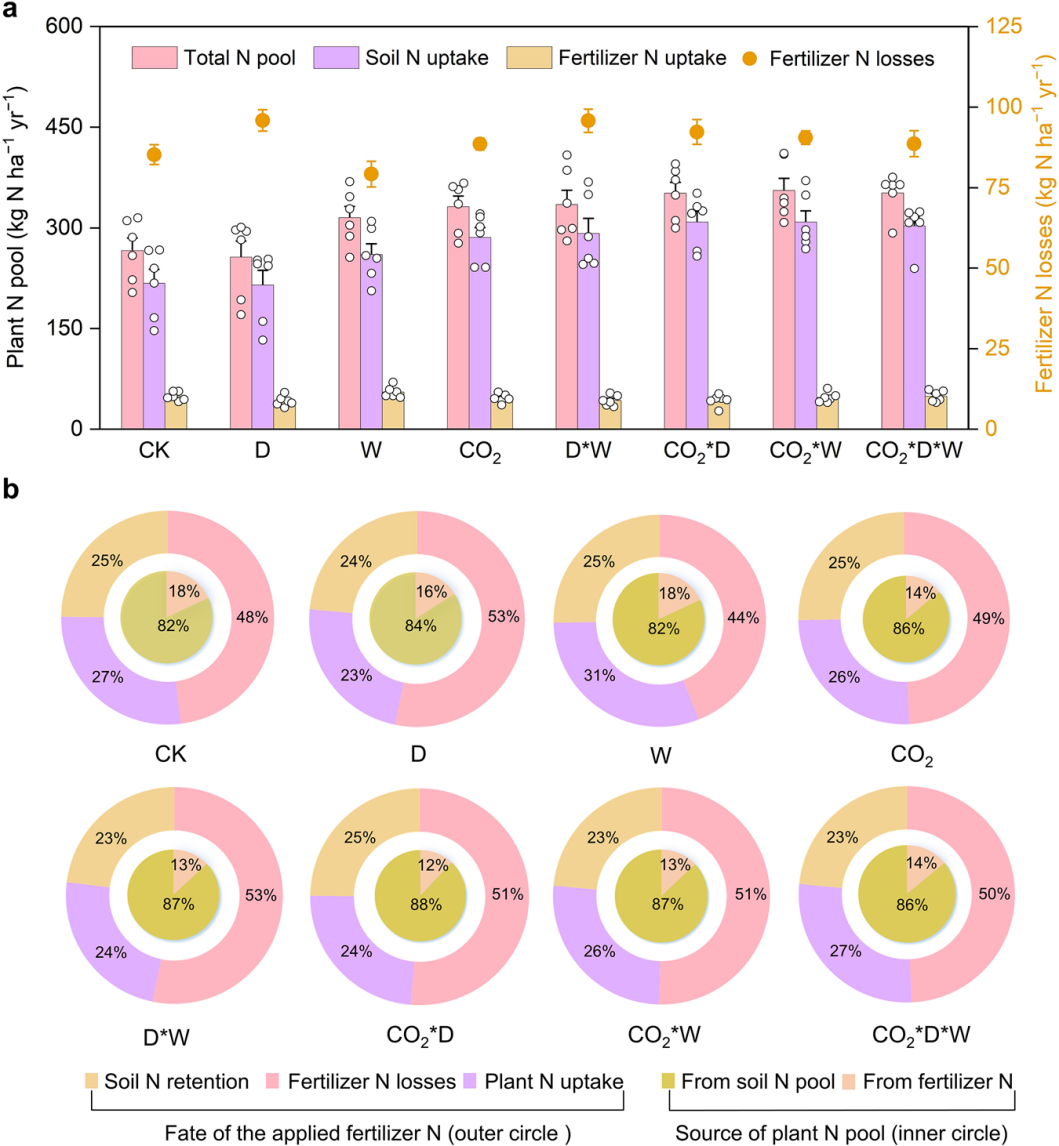
Plant N uptake (a) and fate of applied fertilizer N (b) in different climate change manipulation treatments. Values are two-year averaged from 2020 to 2022. Total N pool, total plant (shoot and root) N uptake; Soil N uptake, plant N uptake from soil N pool; Fertilizer N uptake, plant N uptake from fertilizer N; Soil N retention, soil retention of fertilizer N. W, warming; D, drought; CO_2_, elevated CO_2_ concentration. In **a**, bars show means ± SE and dots represent biological replicates (*n* = 6). Source data are provided as a Source Data file.

**Fig. 3:**
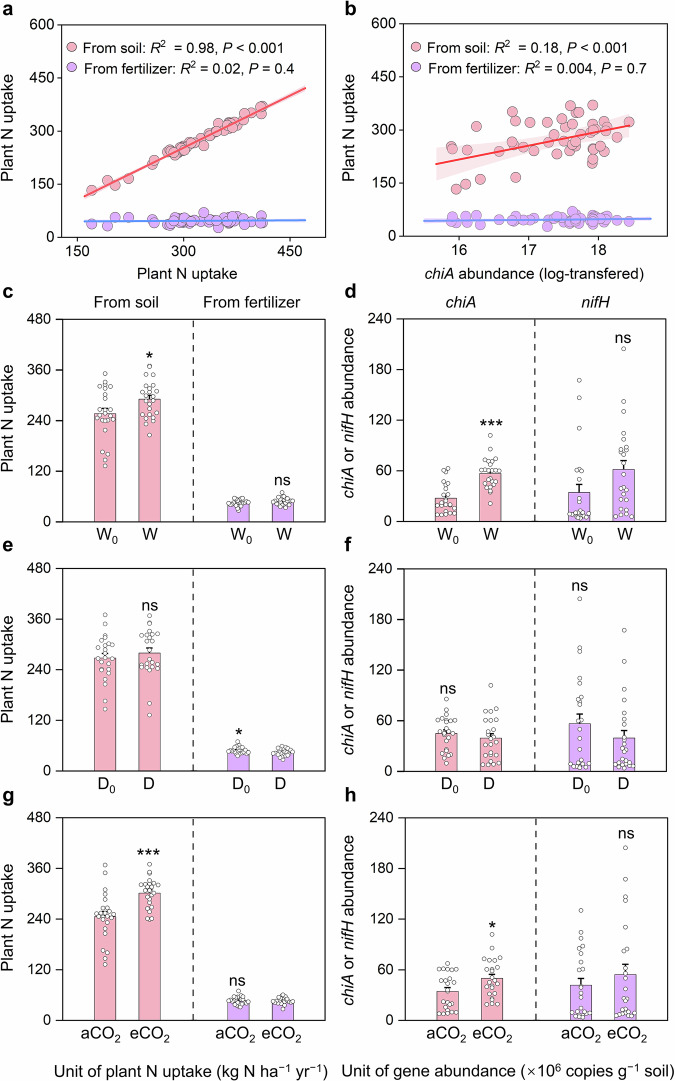
Response of plant N uptake from soil and fertilizer and the associated N decomposition (*chiA*) and fixation (*nifH*) gene abundance to warming, drought and eCO_2_. Values are two-year averaged from 2020 to 2022. Relationship between N uptake from soil/fertilizer with total plant N uptake **a** and *chiA* gene abundance **b**. Effects of warming, drought and eCO_2_ on plant N uptake from soil and fertilizer N **c,** **e**, **g**, and *chiA* and *nifH* gene abundance **d**, **f**, **h**. W_0_, ambient temperature; W, warming; D_0_, ambient rainfall; D, drought; aCO_2_, ambient CO_2_ concentration; eCO_2_, elevated CO_2_ concentration. Shadows denote the 95% confidence intervals for the regression lines **a**, **b**. Values are recalculated across all levels of the other treatments to assess the effects of single climate change factor **c**−**h**. Bars show means ± SE **c**−**h**. Significant differences between groups were determined using one‑way ANOVA followed by a two‑sided Tukey HSD test (*0.01 ≤ *P* < 0.05, **0.001 ≤ *P* < 0.01, ***P < 0.001, ^ns^0.05 ≤ *P*). The exact *P* values of statistical tests are provided in the Source Data file. Dots represent biological replicates (**c**−**h**, *n* = 24). Source data are provided as a Source Data file.

**Fig. 4:**
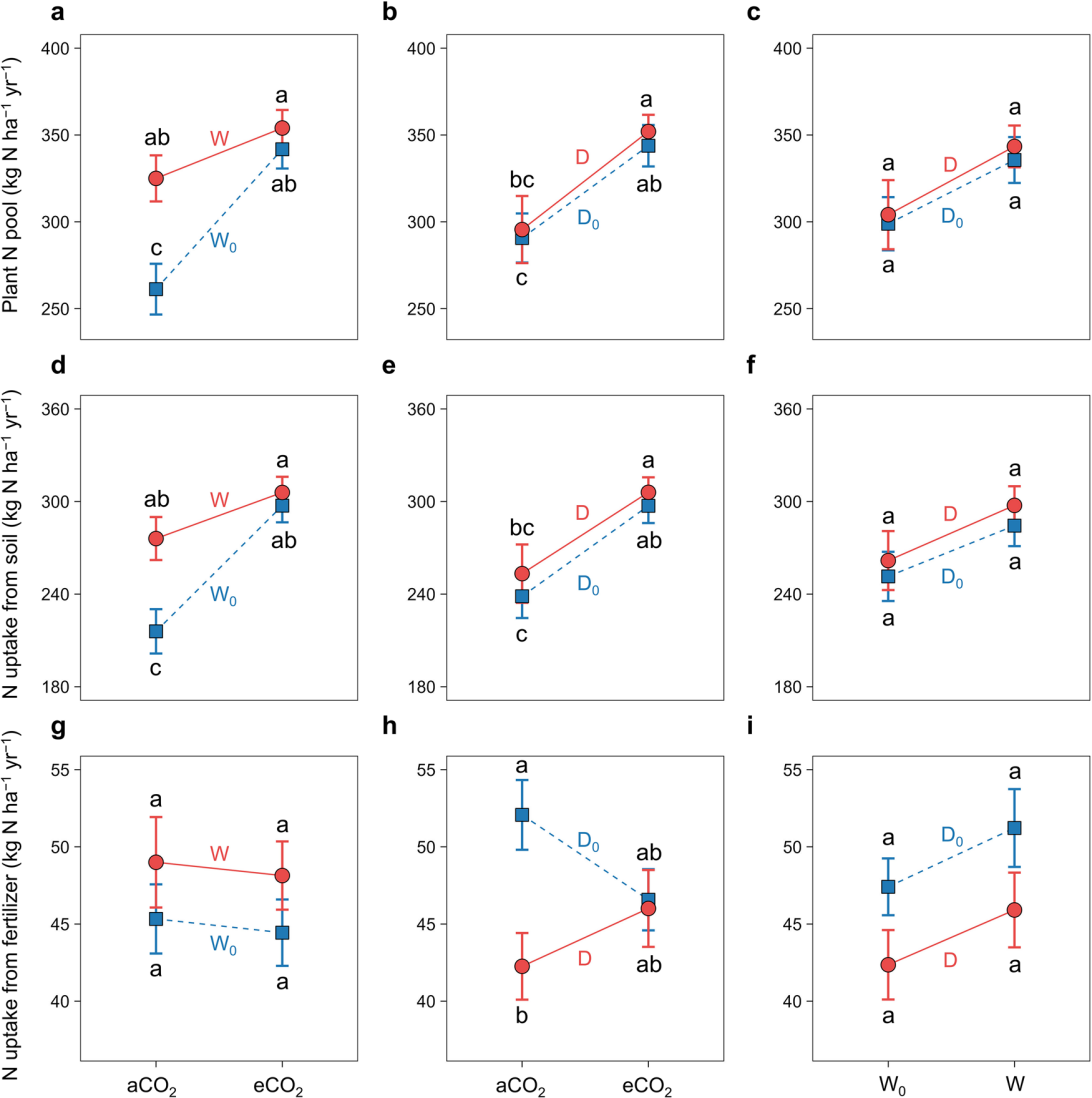
Plant N uptake (a − c) and the associated soil (d − f) and fertilizer N (g − i) contribution in relation to pairs of climate change factors. Values are two-year averaged from 2020 to 2022. W_0_, ambient temperature; W, warming; D_0_, ambient rainfall; D, drought; aCO_2_, ambient CO_2_ concentration; eCO_2_, elevated CO_2_ concentration. Values are recalculated across all levels of the other treatments to assess the effects of combined climate change factors. Bars show means ± SE (*n* = 12, biological replicates). Significant differences between groups were determined using one‑way ANOVA followed by a two‑sided Tukey HSD test. Different letters (e.g., a, b, c) denote statistically significant differences between group means at *P* < 0.05.The exact *P* values of statistical tests are provided in the Source Data file. Source data are provided as a Source Data file.

Climate drivers altered plant N pools in ways that diverged from biomass responses (Figs. 2–3). Over two years, eCO_2_ and warming increased shoot N pool, representing N export by harvest, by 22.9% and 25.7%, respectively (*P* < 0.01), while root N pool remain unaffected (Supplementary Fig. 3). In contrast, drought decreased shoot N pool by 15.1% (*P* < 0.05) but increased root N pool by 28.9% (*P* < 0.05). Interactive effects of climate drivers on shoot N pool was predominantly antagonistic. Responses of root N pool were more variable, exhibiting a mixture of additive, antagonistic and synergistic effects (Supplementary Table 6). Total plant N pool, ranged from 256 to 356 kg N ha⁻^1^ yr⁻^1^ (Fig. 2a), was increased marginally under drought but greatly stimulated by 12.6–35.5% (*P* < 0.05) under warming, eCO_2_, and their combination with drought (Fig. 4; Supplementary Table 7).

Using in-situ ^15^N tracing techniques, we quantified the relative contribution of fertilizer-derived and soil-derived N to plant uptake. Across all treatments, fertilizer-derived N accounted for only 12–18% of total plant N, whereas SON mineralization supplied 215–309 kg N ha⁻^1^ yr⁻^1^ contributing 82–88% of plant N uptake (Fig. 2). Thus, soil-derived N inputs were 4.6–7.3 times greater than fertilizer-derived N, demonstrating a dominant reliance on internal soil N sources. Importantly, N export by harvest under eCO_2_, warming and their combination (182–241 kg N ha⁻^1^ yr⁻^1^) exceeded annual fertilization input (180 kg N ha⁻^1^ yr⁻^1^) (Fig. 2 and Supplementary Fig. 3), providing evidence of net soil N mining under climate change conditions.

Climate change primarily altered plant N acquisition by enhancing uptake from soil rather than fertilizer sources. Specifically, warming and eCO_2_ significantly increased total plant N acquisition from soil N pools by 13.4% (*P* < 0.05) and 22.6% (*P* < 0.001), respectively (Fig. 3c,g). Drought exerted contrasting organ-level effects, but increased root N uptake from SON ( + 28.9%, *P* < 0.05), which offset reduced shoot uptake (–15.1%, *P* < 0.05), resulting in a small net increase in total plant N acquisition from soils by 4.4% (*P* > 0.05; Supplementary Table 7).

Combined climate treatments further amplified soil-derived N uptake, with the strongest increase (+41.7%, *P* < 0.001) observed under eCO_2_×warming (Fig. 4; Supplementary Table 7). Interactive effects were predominantly antagonistic for eCO_2_×warming and eCO_2_×drought, but synergistic for warming×drought (Fig. 4). In contrast, fertilizer-derived N uptake by plants (N use efficiency, 23–31%) remained largely unchanged across all treatments (Figs. 3–4), suggesting limited responsiveness of plant access to external N inputs under climate change. Most applied fertilizer N was lost to the environment (47–53%), while only 23–25% was remained in soil (Fig. 2). Among climate drivers, drought significantly increased fertilizer N losses (*P* < 0.01; Supplementary Fig. 3), whereas warming and eCO_2_ had no detectable effects on fertilizer N losses or soil N retention (Fig. 2; Supplementary Fig. 4).

### Climate change accelerates depletion of soil nitrogen pools

**Fig. 5:**
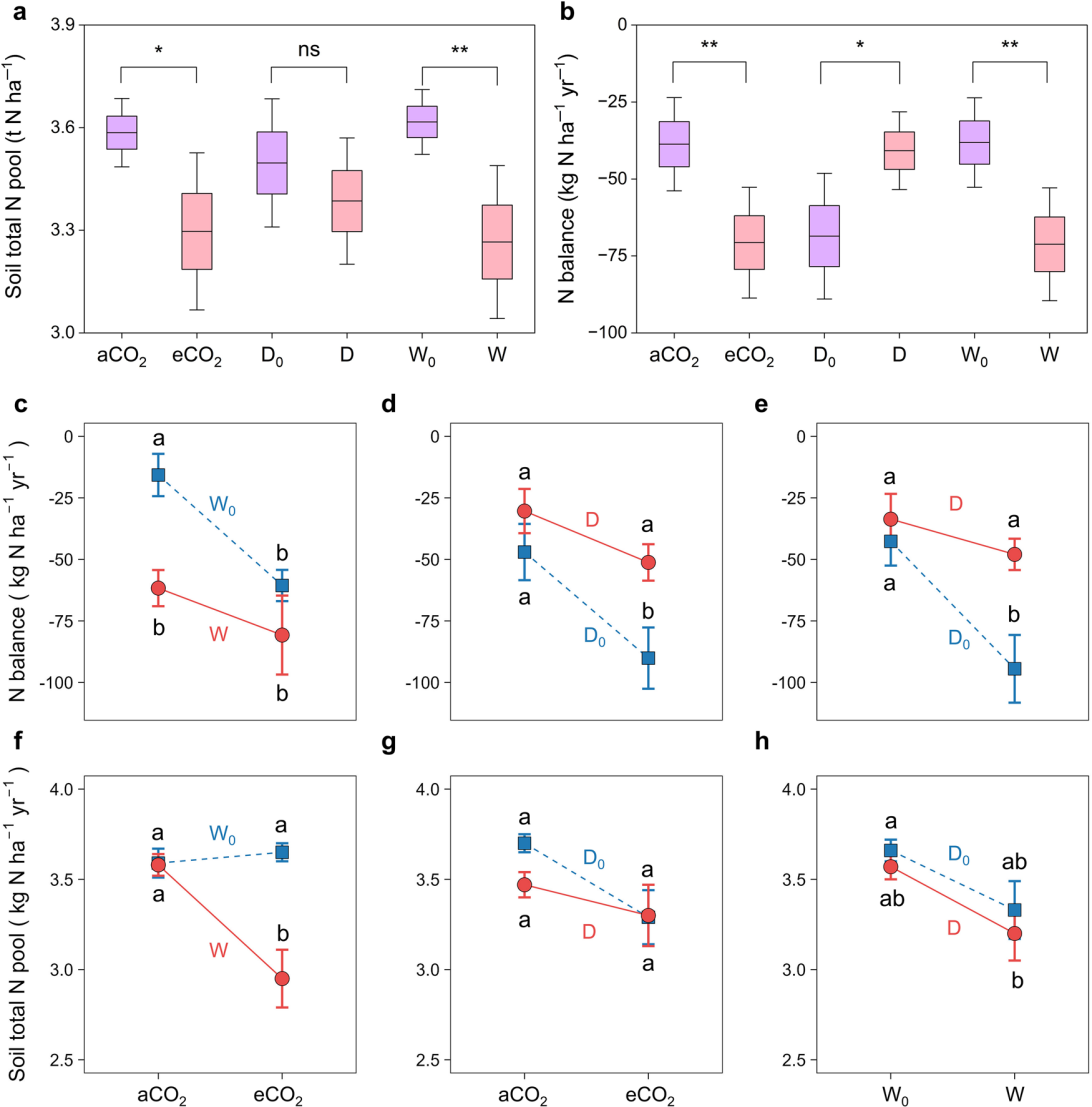
Soil total N pool and soil-plant N balance in single (a − b) and interactive (c − h) climate change factors. W_0_, ambient temperature; W, warming; D_0_, ambient rainfall; D, drought; aCO_2_, ambient CO_2_ concentration; eCO_2_, elevated CO_2_ concentration. Negative values of N balance denote net soil N depletion. Values are recalculated across all levels of the other treatments to assess the effects of single and combined climate change factors. In **a** − **b**, the whiskers of the boxes illustrate the 5^th^ and 95^th^ percentiles and the central line represent the mean values (*n* = 24, biological replicates). In **c** − **h**, bars show means ± SE (*n* = 12, biological replicates). Significant differences between groups were determined using one‑way ANOVA followed by a two‑sided Tukey HSD test (*0.01 ≤ *P* < 0.05, **0.001 ≤ *P* < 0.01, ***P < 0.001, ^ns^0.05 ≤ *P*). Different letters (e.g., a, b, c) denote statistically significant differences between group means at *P* < 0.05. The exact *P* values of statistical tests are provided in the Source Data file. Source data are provided as a Source Data file.

**Fig. 6:**
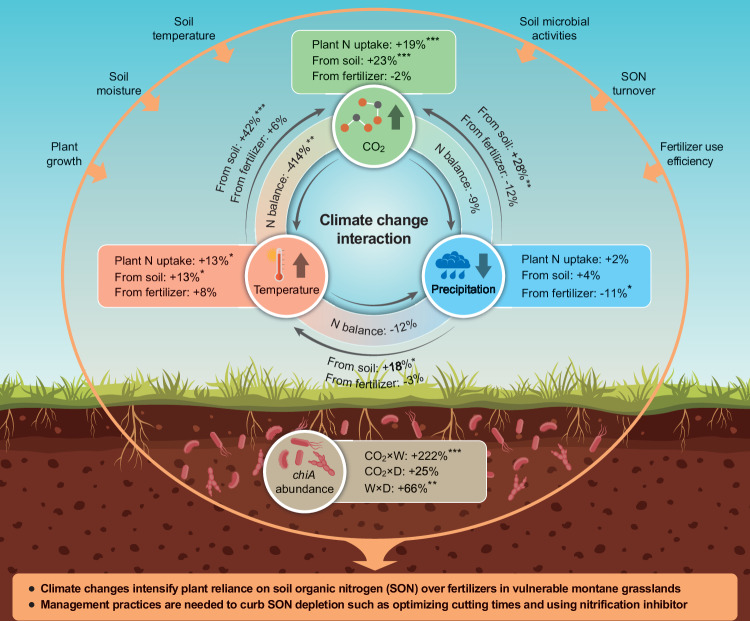
Overall effects of climate change on soil-plant N cycles in montane grassland ecosystems. Plant N uptake, total shoot and root N uptake; From soil, plant N uptake from soil N pool; From fertilizer, plant N uptake from fertilizer N; SON, soil organic N pool; NUE, fertilizer N use efficiency. W, warming; D, drought; CO_2_, elevated CO_2_ concentration. Significant differences between groups were determined using one‑way ANOVA followed by a two‑sided Tukey HSD test (*0.01 ≤ *P* < 0.05, **0.001 ≤ *P* < 0.01, ***P < 0.001, ^ns^0.05 ≤ *P*).

Ecosystem N balances revealed sustained losses of soil N across all treatments. Combining fertilizer inputs, plant N export, and estimated fertilizer N losses (based on unrecovered ^15^N) (see Methods), we found that total N output by plant harvest and N losses consistently exceeded fertilizer inputs, resulting in consistently negative ecosystem N balances from –94.5 to –15.7 kg N ha⁻^1^ yr⁻^1^ (Fig. 5). These deficits suggest net depletion of SON pools. Relative to ambient conditions, warming, eCO_2_, and their combinations with drought further reduced ecosystem N balances by 4.2–65 kg N ha⁻^1^ yr⁻^1^ (9.0–414%; Fig. 5; Supplementary Table 7). Accordingly, total soil N pools declined by 3.2–17.8% under individual and combined climate treatments compared to ambient controls (Fig. 5). Notably, multifactorial climate treatments caused stronger soil N depletion than any single driver, with the largest decline occurring under combined eCO_2_ and warming (Fig. 6). These results demonstrate that interacting climate drivers accelerate soil N depletion, reinforcing the dominance of soil-derived N in plant nutrition while progressively exhausting the soil N reservoir.

## Discussion

### Climate change regulates plant N acquisition through distinct physiological and allocation pathways

Elevated CO_2_ is widely recognized for stimulating plant photosynthesis and biomass production^24,25^. Consistent with this, we observed a 24–35% increase in shoot and root biomass (Fig. 1), aligning with global syntheses reporting 19–34% stimulation^10^. This biomass enhancement was accompanied by a decline in shoot N concentration, reflecting the well-established growth dilution effect^12,26^. However, increased biomass more than compensated for the reduced tissue N concentrations, resulting in a significant rise in total plant N pools (Fig. 4). Warming influenced plant N acquisition primarily through enhanced soil N supply rather than changes in biomass allocation. Despite a higher shoot-to-root ratio (Supplementary Table 2), plant N pools increased under warming, indicating that accelerated soil N mineralization played a dominant role^14,27^. This interpretation is supported by the significantly increased abundance of the *chiA* gene (Fig. 1 and Supplementary Fig. 5), which encodes a chitin-degrading enzyme involved in N release from soil organic matter^28^. In contrast, drought promoted root allocation, as indicated by a reduced shoot-to-root ratio (*P* < 0.05; Supplementary Table 3), reflecting an adaptive response to water limitation^13,19^. Increased root N uptake offset declines in shoot N acquisition, resulting in a slight net increase in total plant N pools under drought.

When combined, climate drivers increased total plant N pools by 14.9–35.5% (Fig. 4), but their effects were largely non-additive. While previous studies have often reported additive responses of biomass and N accumulation^1,12,29^, we observed predominantly antagonistic interactions for root biomass, tissue N concentrations, and plant N uptake (Fig. 4; Supplementary Tables 6–7), with additive effects observed only for shoot biomass. These patterns likely arise because warming-induced increase in soil N mineralization reduced the need for eCO_2_-driven root proliferation for nutrient acquisition^26^, thereby constraining root biomass under combined eCO_2_ and warming relative to eCO_2_ alone. In addition, the eCO_2_-induced growth dilution effect may offset warming-enhanced N uptake^15,30^, resulting in a smaller-than-expected increase in the total plant N pool under combined treatment. Interactions involving drought were more variable. For example, warming and drought showed synergistic effects on root biomass and N uptake, likely reflecting increased carbon allocation to roots for water acquisition^31,32^. These non-linear interactions highlight the difficulty of predicting ecosystem responses to concurrent climate drivers, particularly in montane grasslands where additive assumptions remain common^2,33,34^.

### Soil nitrogen, not fertilizer, sustains plant productivity under climate change

A central finding of this study is the dominant role of soil-derived N in sustaining plant growth (Fig. 6). Across all treatments, 82–88% of plant N originated from SON pool, with fertilizer contributing only 12–18% (Fig. 2). Despite substantial fertilizer inputs (180 N ha⁻^1^ yr⁻^1^), soil-derived N exceeded fertilizer-derived N by up to sevenfold, and plant N pools were strongly correlated with soil-derived N (*P* < 0.001) but not with fertilizer-derived N (Fig. 3a). This decoupling reflects the inherently low efficiency of slurry-based fertilization systems^35–37^. Liquid slurry is the predominant fertilizer form in pre-Alpine grasslands^21,38^, which is typically broadcast across fields^39^. Approximately 47% of the applied N was lost as gaseous emissions (e.g., ammonia and dinitrogen^22^), while only about a quarter was retained in soils, resulting in a fertilizer N use efficiency (NUE) of ~25% (Fig. 2). Such low NUE was insufficient to meet plant N demand (266 kg N ha⁻^1^ yr⁻^1^), necessitating continuous mineralization of SON to sustain productivity^21^. As a result, current management practices effectively shift the burden of plant nutrition onto soil N reserves.

Strikingly, climate change did not alter the contribution of fertilizer-derived N to plant uptake. This contrasts with expectations that eCO_2_ or warming would enhance fertilizer use efficiency and buffer ecosystems against N limitation^5,12,40^. Following slurry application, liquid N was rapidly immobilized by microbes or lost as gaseous emissions^23,41^, while the solid fraction remained on the soil surface with limited microbial access. The slow nutrient release from the solid fraction was therefore insufficient to meet rising plant N demands under eCO_2_^21^, which instead stimulated root foraging for acquiring soil-derived N^20^. Visual observations during the experiment confirmed that, under warming and drought conditions, the solid fraction may have dehydrated quickly and formed a dry crust, potentially restricting microbial breakdown, making it even more difficult for plants to access N from the crust^39^. Consequently, climate changes have minimal impact on the contribution of fertilizer N to plant N uptake.

In contrast, soil N cycling exhibited strong sensitivity to climate drivers. Warming and eCO_2_ enhanced microbial activity and accelerated SON mineralization^42^, which increased soil N availability and plant uptake^42–44^. Enhanced microbial assimilation of fertilizer-derived ammonium (NH_4_^+^) may induce stoichiometric imbalance and stimulates SON mineralization to satisfy microbial and plant N uptake, consistent with the microbial N pump mechanism^45^. In addition, eCO_2_-induced increases in root exudation, combined with slurry-derived carbon inputs, likely further stimulated microbial decomposition of soil organic matter, intensifying microbial N mining. Although gene abundance does not necessarily directly reflect microbial process rates, the consistent increase in *chiA* abundance (45–106%), together with enhanced soil-derived plant N uptake, supports the inference that warming and eCO_2_ stimulated microbial decomposition and SON mobilization (Fig. 3). By contrast, drought imposed water limitation on microbial process^46,47^, suppressing soil N transformation rates^47^, and reducing the supply of soil-derived N to aboveground plant tissues. However, this decline was partly offset by increased root N pools, resulting in a non-significant net increase in total plant N derived from soil (Fig. 4). Nevertheless, eCO_2_ and warming override drought effects, leading to consistent increases in soil-derived N acquisition across all multifactor climate treatments (Supplementary Tables 6–7). Collectively, these results demonstrate that climate change significantly strengthens plant reliance on soil-derived N, even under intensive organic N fertilization, in montane grassland ecosystems.

### Accelerated soil nitrogen depletion under future climates and implications for nitrogen management

The increasing reliance on soil-derived N has direct consequences for ecosystem N balance. Across all treatments, ecosystem N budgets showed persistent deficits, with N outputs through harvest and losses consistently exceeding fertilizer inputs (Fig. 5). This imbalance indicates that plant productivity was maintained through net depletion of soil N pools rather than external organic N fertilization inputs. Although eCO_2_ has been proposed to increase ecosystem N stocks via enhanced root exudation and litter inputs^10^, our results showed that these gains were outweighed by increased N export and losses^48^, resulting in an overall decline in soil total N pools (–8%) (Fig. 5). Moreover, we found no evidence for compensatory biological N fixation, as indicated by unchanged *nifH* gene abundance (Fig. 3h). Warming further accelerated soil N depletion, causing a 9.7% decline in soil N pools (Fig. 5), greater than the reductions reported for temperate and tropical ecosystems^8,49,50^. This result suggests that montane grassland soils are particularly sensitive to temperature increases. Although drought reduced soil N turnover, rewetting events stimulated microbial activity^13,47^, and exacerbated N losses (Supplementary Fig. 3), resulting in a net decline in soil N (–3.2%). More concerningly, multifactor climate treatments amplified soil N depletion compared to ambient conditions, with the greatest losses under combined eCO_2_ and warming (–17.8%). These results reveal a reinforcing feedback in which climate change increases plant N demand and accelerates soil N mobilization, leading to progressive depletion of soil N pools—a process we term climate-driven soil N mining in organically fertilized grassland ecosystems. A recent study in rice-cropping systems similarly showed that chemical N fertilization can stimulate SON mineralization and promote soil-derived N losses and plant N uptake via the microbial N pump mechanism^45^, suggesting that climate-driven soil N mining may represent a more general feature of intensively fertilized agroecosystems.

These findings highlight the urgent need to improve N management in grassland systems to tackle with climate change (Fig. 6). Current slurry-based fertilization practices are insufficient to offset increasing plant N demand and ongoing soil N depletion. Without intervention, continued reliance on these practices will likely compromise long-term ecosystem productivity. Improving fertilizer efficiency is therefore critical^51,52^. Incorporating slurry into soils, rather than surface application, can substantially increase NUE (by 21.5–42.6%) and reduce N losses (by 14.6–38.6%)^51,53^, while maintaining plant productivity. The use of nitrification inhibitors can also effectively enhance the contribution of fertilizer-derived N to plant N pool and alleviate soil N depletion^54^. In addition, reducing harvest intensity is essential, as frequent cutting (~4 times per year) drives substantial N export and accelerates soil N depletion. More extensive management practices, such as reducing cutting frequency, have been shown to significantly lower soil N mineralization rates in montane grasslands^17^. However, the effectiveness of these strategies under future climate conditions was not directly tested in this study. Given the strong sensitivity of soil N cycling to climate drivers, their effectiveness under future climate change requires further experimental validation. Long-term, integrative studies linking plant N uptake, soil N transformations, and N losses are essential to improve predictive models and guide sustainable management strategies under a rapidly changing climate.

Several uncertainties associated with our experimental approach should be acknowledged. First, the ^15^N-labeling strategy primarily targeted the dominant readily plant-available N forms in the slurry (NH_4_⁺-N and urea-N), which together accounted for the vast majority of total slurry N (see Methods). A small fraction of particulate organic N remained unlabeled and may have been partially taken up by plants without being traced in the fertilizer-derived N pool. As a result, estimates of fertilizer N recovery likely represent a conservative bound, with a minor potential bias toward underestimating fertilizer-derived uptake and overestimating soil-derived N. However, this is unlikely to affect our central conclusion that plant N uptake was overwhelmingly dominated by soil-derived N across treatments. Second, biological N fixation was estimated using empirical relationships and assumed to be invariant among treatments, consistent with the absence of significant differences in *nifH* gene abundance under climate manipulation. Although this introduces uncertainty into ecosystem N budgets, BNF contributed only a minor fraction of total N inputs relative to slurry fertilization. Finally, because the experiment focused on slurry-based organic fertilization, observed responses may partly reflect interactions between climate change and slurry-derived carbon inputs. Future work should test whether similar patterns of climate-driven soil N mining occur under mineral fertilizer systems. Nevertheless, these uncertainties do not alter the main conclusion that climate change intensified plant reliance on soil-derived N despite intensive organic N fertilization.

## Methods

### Study sites and experimental design

This study was established as a sub-experiment of grassland research conducted in the TERENO Pre-Alpine Observatory^11^. The experiment employed a full-factorial design comprising eight treatment combinations and 12 replicates, manipulating three key climate factors: temperature (ambient vs. +2.5°C warming), atmospheric CO_2_ concentration (ambient vs. +300 ppm), and growing-season precipitation (ambient vs. −30% from April to October) (Supplementary Table 1). These levels were selected to represent projected climatic conditions for Central Europe under intermediate- to high-emission scenarios by the late 21st century. The elevated CO_2_ treatment approximates concentrations expected under SSP2-4.5 to SSP3-7.0 scenarios, while the +2.5 °C warming reflects regional temperature increases projected for the same range of scenarios^55^. The 30% reduction in growing-season precipitation represents current-to-projected seasonal water deficits and increasing drought frequency in the pre-alpine region.

In June 2020, a total of 100 undisturbed plant–soil mesocosms (8 treatments × 12 replicates, and 4 held in reserve) were collected using stainless-steel cylinders (17 cm diameter × 25 cm height; open at both ends with a sharpened lower edge) from grasslands adjacent to the IMK-IFU campus (47°28′N, 11°04′E; 720 m above sea level), with a mean annual temperature and mean growing season temperature of 8.2 °C and 10.6 °C, respectively, and annual precipitation of 1,386 mm (mean of 2012–2021). The soils are classified as Calcaric Cambisol, with a top soil (0–15 cm) bulk density of 0.35 g cm⁻^3^, pH of 6.83 and organic carbon and total N contents of 53.5 g Ckg⁻^1^ and 6.3 g N kg⁻^1^ SDW, respectively. The dominant grass species of the montane grassland are *Lolium perenne L*. and F*estuca rubra L*., forbs including *Plantago lanceolata L*., *Prunella vulgaris L*., *Ranunculus acris L*., and *Carum carvi L*., and legume *Trifolium repens L*.

Experimental treatments were implemented in the IMK-IFU controlled greenhouse facility, which was equipped with UV-transmissive glass and automated environmental controls. In June 2020, all mesocosms were randomly distributed across four PVC boxes (Boxes 1–4) housed within two chambers (Chambers A and B). Each box contained 25 mesocosms (Supplementary Fig. 1). Chamber A received ambient CO_2_ ( ~ 465 ppm), while Chamber B was enriched to ~784 ppm during daytime hours (08:00–18:00) throughout the growing season (April–November). The control of climatic conditions and CO_2_ concentrations in the separate chambers of the greenhouse facility has been described before^56,57^. Air temperature in both chambers was maintained ca. 2.5°C above outdoor levels while relative humidity was maintained at 13.5 mmol/mol and air pressure was 92.6 kPa. For non-warming treatments a cooling system using stainless-steel tubes with circulating cold water was installed in Boxes 2 and 3 to offset the temperature increase (Supplementary Fig. 1), thereby maintaining ambient soil temperatures in those boxes. Consequently, Boxes 1 and 4 represented the warming treatments (+2.5°C), and Boxes 2 and 3 served as ambient temperature controls. Mesocosms were embedded in sand within each box to match the soil surface level, and the sand was regularly irrigated to maintain a stable soil temperature control. Soil temperature and moisture in the mesocosms were recorded every 5 minutes (SMT-100 sensors, UGT, Germany) in one representative mesocosm per treatment.

Historical climate (2012–2021) records from a nearby weather station of the German Weather Service indicate that droughts in the study region typically reduce annual precipitation by ~30%, with spring and autumn being the drier seasons. As the greenhouse excluded natural rainfall, irrigation was applied manually using syringes, with timing and quantity adjusted according to actual precipitation events. Drought was imposed by excluding irrigation each for a three-week period in spring (April–May) and autumn (August–September)—followed by natural rewetting to ensure plant survival and comparable productivity across treatments. Climate manipulations were applied during the growing seasons (April–November) of 2020–2022. During winter (December–March), all mesocosms were placed outside with the mesocosms buried into an adjacent montane grassland to ensure exposure to natural cold and snowpack conditions (Supplementary Fig. 1). The eight treatment combinations included: ambient control (CK), drought (D), warming (W), elevated CO_2_ (eCO_2_), drought×warming (D×W), eCO_2_×drought (eCO_2_×D), eCO_2_×warming (eCO_2_×W), and eCO_2_×drought×warming (eCO_2_×D×W) (Fig. 2). Of the 12 mesocosm set up per treatment, six were used for plant biomass and N measurements, and six for soil sampling. Over the two growing seasons, average soil temperatures in the ambient and warmed treatments were 9.5°C and 12.2°C, respectively, while average soil moisture was 34.9% under ambient precipitation and 29.6% under drought (Supplementary Table 1).

### ^15^N tracing approach

The studied montane grasslands were intensive managed and underwent 4 cuttings and subsequent 4 times fertilizations per year. In line with regional agricultural practices, ~180 kg N ha⁻^1^ per year cattle slurry was surface applied to the studied plant-soil mesocosms, with each single slurry fertilization contributing approximately 45 kg N ha⁻^1^. The slurry was usually applied in mid-April, early-July, end-August and end-October. We conducted ^15^N tracer techniques to assess soil–plant N dynamics in response to the three factorially manipulated climate change drivers—eCO_2_, warming and drought. The ^15^N labeling of the mesocosms was conducted via the application of ^15^N enriched liquid cattle slurry. The cattle slurry used for the experiment was obtained from a local farmer and analyzed for N and nutrient content by a commercial laboratory (Raiffeisen Laborservice, Ormont, Germany). The used slurry had a pH of ~7.5 and a total nitrogen content of ~2.0 g N kg⁻^1^ fresh weight (FW), comprising ~0.2 g N kg⁻^1^ FW unextractable organic N, and ~1.8 g N kg⁻^1^ FW dissolved N, including ammonium (NH_4_⁺-N) and urea-N. The time line of the management practice (e.g., fertilization and harvesting) was shown in Supplementary Fig. 1.

Based on these analyses, the intended ^15^N enrichment of the slurry was set to ca. 5% and executed via the addition of 99% ^15^N enriched ammonium sulfate and urea in equal N amounts to mimic fresh cattle slurry and label more than four-fifths of N forms in the slurry. Immediately before each application, the ^15^N tracer was added to the slurry in a polyethylene barrel and vigorously shaken to ensure a homogenous mixture with the slurry. Each mesocosm was fertilized with an amount of 40 ml liquid manure per fertilization event, representing farmers practice with an application rate of 1.8 m^3^ ha^−1^. This resulted in an addition of 5.7 mg ^15^N in excess of natural abundance per mesocosm and fertilization event. Addition of ^15^N label marginally increased the N content of slurry. Because a small fraction of slurry organic N was not uniformly labeled, the recovery-based estimates of fertilizer-derived N may be slightly underestimated, with a corresponding slightly overestimation of soil-derived N. However, as the majority of plant-available N forms in the slurry was labeled and the tracer enrichment was high, this potential bias (<5%) is minimal. Recovery calculations were based on the actual ^15^N input, ensuring that the inherent uncertainty associated with unlabeled slurry N does not affect the central conclusion that plant N uptake is dominated by soil-derived sources under climate change.

### Biomass harvesting, soil sampling and analysis

Aboveground biomass was harvested following local management practices by cutting vegetation 5 cm above the soil surface using scissors before slurry application. Harvested biomass was placed in paper bags and transported to IMK-IFU’s lab for further processing. Biomass was initially air-dried by turning it every two days for one week, then oven-dried at 60°C to constant weight. Dried material was weighed, finely chopped, homogenized, and a representative subsample was ground using a ball mill (Retsch MM301, Germany) at 30 rpm for 3 minutes. Destructive soil sampling was conducted in the end of the experiment (August 2022). Soil samples were collected from each mesocosm at three depths (0–5 cm, 5–15 cm 15–25 cm). The soil samples were sieved by using a 2 mm mesh, dried at 60 °C until a constant weight was achieved, and ground using a ball mill. For sampling of roots from a defined volume, we used separately sampled cores of 100 cm^3^ volume for three depths. Roots were handpicked and washed with distilled water. The roots were immersed in a capacious container or sink filled with water and swirled gently to eliminate any residual soil. Finally, the roots were dried and pulverized as described above. Aliquots of biomass (2 mg), root (4 mg), and soil samples (2–8 mg) were weighed into 5×9 mm tin capsules (IVA Analysentechnik, Meerbusch, Germany). Total N concentration and ^15^N enrichment were determined at IMK-IFU Center for Stable Isotopes using an elemental analyzer (Flash 1112 EA, Thermo Scientific, USA) coupled to an isotope ratio mass spectrometer (Delta Plus XP, Thermo Scientific, USA) via a ConFlo III interface. For soil microbial characterization, the abundances of the *nifH* and *chiA* genes for soils samples collected after the final harvest (July 2022) were quantified using quantitative real-time PCR (qPCR) following protocols by Wang et al^21^. and Tuner et al^58^., which were described in detail in the Supplementary Information (Supplementary Fig. 5).

### Quantification of fertilizer- and soil-derived N, N losses, retention, and ecosystem N balance

The excess ^15^N (mg) in all measured N pools including soil, shoot and root biomass was calculated using the following equation:

1 $${{{\rm{N}}}}_{{{\rm{excess}}}}={{{\rm{N}}}}_{{{\rm{pool}}}}\times ({}^{15}{{\rm{N}}}_{{{\rm{L}}}}-0.3663)/100$$

where N_pool_ is the N content (mg N mesocosm⁻^1^) in the respective pool and ^15^N is the atom% ^15^N. A natural abundance of 0.3663% was assumed, with negligible error due to the high level of ^15^N enrichment in the labeled slurry. Recovery (%) was calculated as the ratio of ^15^N excess in each pool to the cumulative ^15^N input from slurry applications.

The relative contributions of fertilizer-derived and soil-derived N to plant uptake were quantified using a ^15^N isotope dilution approach. Fertilizer-derived N uptake by plants (N_fert_) was determined from the recovery of ^15^N tracer in total plant biomass (shoots + roots), expressed as the proportion of total applied ^15^N recovered in plant pools:

2 $${{{\rm{N}}}}_{{{\rm{fert}}}}={}^{15}{{\rm{N}}}_{{{\rm{plant}}}}/{}^{15}{{\rm{N}}}_{{{\rm{applied}}}}\times {{{\rm{N}}}}_{{{\rm{applied}}}}$$

Where ^15^N_plant_ is the total excess ^15^N recovered in plant biomass, and ^15^N_applied_ is the cumulative ^15^N added via labeled slurry. N_applied_ is the total slurry N application rate (180 kg Nha⁻^1^ yr⁻^1^).

Total plant N uptake (N_total_) was determined from measured plant biomass and N concentrations. Soil-derived N uptake (N_soil_) was then calculated by mass balance:

3 $${{{\rm{N}}}}_{{{\rm{soil}}}}={{{\rm{N}}}}_{{{\rm{total}}}}-{{{\rm{N}}}}_{{{\rm{fert}}}}$$

The proportional contributions (%) of fertilizer- and soil-derived N were calculated relative to total plant N uptake. This approach assumes that ^15^N enrichment in plant tissues originates exclusively from labeled fertilizer inputs, while unlabeled N represents soil-derived sources. Because a small fraction of slurry organic N was not uniformly labeled, fertilizer-derived N may be slightly underestimated and soil-derived N correspondingly slightly overestimated; however, this bias is minimal (<5%) due to the high labeling effectiveness on the slurry N.

The fate of applied fertilizer N was assessed using a ^15^N recovery framework. Total recovery of applied ^15^N was quantified as the sum of ^15^N recovered in plant biomass and soil pools. Slurry-derived N losses (N_loss_) were estimated as the unrecovered fraction of applied ^15^N:

4 $${{\rm{N}}}_{{\rm{loss}}}={{\rm{N}}}_{{\rm{applied}}}-({{\rm{N}}}_{{\rm{fert}}}+{{\rm{N}}}_{{{\rm{soil}}}\_{{\rm{ret}}}})$$

where N_soil_ret_ is the amount of fertilizer-derived N retained in soil, calculated from the recovery of ^15^N in soil pools:

5 $${{{\rm{N}}}}_{{{\rm{soil}}}\_{{\rm{ret}}}}={}^{15}{{\rm{N}}}_{{{\rm{soil}}}}/{}^{15}{{\rm{N}}}_{{{\rm{applied}}}}\times {{{\rm{N}}}}_{{{\rm{applied}}}}$$

Here, ^15^N_soil_ represents the total excess ^15^N recovered in soil. The estimated N losses therefore include all pathways not directly quantified, such as gaseous emissions (e.g., NH_3_, N_2_O, N_2_) and leaching.

Ecosystem N balance was calculated by integrating all major N inputs and outputs:

6 $${{{\rm{N}}}}_{{{\rm{balance}}}}={{{\rm{N}}}}_{{{\rm{input}}}}{{\rm{\hbox{-}}}}{{{\rm{N}}}}_{{{\rm{output}}}}$$

where total N inputs include slurry N application, atmospheric N deposition, and biological N fixation (BNF), and outputs include plant N export via harvest and slurry-derived N losses. Specifically:

7 $${{{\rm{N}}}}_{{{\rm{balance}}}}=\,{{{\rm{N}}}}_{{{\rm{applied}}}}+{{{\rm{N}}}}_{{{\rm{deposition}}}}+{{{\rm{N}}}}_{{{\rm{BNF}}}}{{\rm{\hbox{-}}}}{{{\rm{N}}}}_{{{\rm{harvest}}}}{{\rm{\hbox{-}}}}{{{\rm{N}}}}_{{{\rm{loss}}}}$$

Atmospheric N deposition was set to 16 kg N ha⁻^1^yr⁻^1^ due to winter snow falls, based on regional monitoring in Bavarian pre-alpine grasslands^21^. As the abundance of the *nifH* gene did not significantly differ among treatments (Fig. 3; Supplementary Fig. 5), BNF was assumed to be unaffected by climate manipulation. BNF was estimated using observed legume abundance (3% of total biomass) and established empirical relationships from comparable grasslands, yielding an average BNF rate of 15 kg N ha⁻^1^yr⁻^117^. We acknowledge that this approach may introduce uncertainty because the studied grassland was intensively managed with slurry application. However, legumes constituted only a small fraction of total biomass, and BNF contributed minimally to total ecosystem N inputs relative to slurry fertilization. Plant N export (N_harvest_) was determined from measured shoot biomass and N concentration. Negative N balance values indicate net depletion of soil N pools. Although ecosystem N balances were estimated using currently available regional data and empirical relationships, the resulting ecosystem N balances should be interpreted as indicative of relative treatment effects rather than precise ecosystem-scale N budgets.

All data were tested for normality (Kolmogorov–Smirnov test) and homogeneity of variance (Levene’s test). Significant differences between groups were determined using one‑way ANOVA followed by a two‑sided Tukey HSD test. Interactive effects among climate change factors were quantified using a repeated measures mixed model with both fixed and random effects^2^. Calculations for two- and three-way interactions were done across all levels of the other treatments. Expected effects were the additive effects sizes of each treatment when imposed individually (Supplementary Tables 6–7). Synergistic interactions were defined where observed responses exceeded expected values, and antagonistic interactions where they were lower^2^.

### Reporting summary

Further information on research design is available in the Nature Portfolio Reporting Summary linked to this article.

## Supplementary information

Peer Review file

Supplementary Information

Reporting Summary

## Source data

Source Data

## Data Availability

The study data are included in the article and/or Supplementary documents. Source data are provided with this paper.

## Code availability

This study does not generate codes.
