# Supplementary material for: Elevated CO2 and warming intensify plant reliance on soil nitrogen reserves despite intensive fertilization
Source: Nat Commun. 2026 Jul 8;17:5979. doi: 10.1038/s41467-026-75147-w (PMC13346951; doi:10.1038/s41467-026-75147-w)
Supplement: Supplementary file 2 — Supplementary Information [file 41467_2026_75147_MOESM2_ESM.pdf]

Supplementary Information for

Elevated CO_2 and warming intensify plant reliance on soil nitrogen reserves despite intensive fertilization

Yuhao Zhu[1,2#], Li Wan[1,3#], Longlong Xia[1,4*], Klaus Butterbach-Bahl[5,1], Michael Dannenmann[1], Clemens Scheer[1], Nadine K. Ruehr[1], Benjamin Wolf[1], Xiaoyuan Yan[4], Zhijun Wei[4], Peter B. Reich[6,7], Yiqi Luo[8], Pete Smith[9], Josep Peñuelas[10], Michael Schloter[11], Stefanie Schulz[11], Ralf Kiese[1]

[1] Institute for Meteorology and Climate Research (IMK-IFU), Karlsruhe Institute of Technology, Garmisch-Partenkirchen 82467, Germany.

[2] Key Laboratory of Mountain Surface Processes and Ecological Regulation, Institute of Mountain Hazards and Environment, Chinese Academy of Sciences, Chengdu 610213, China.

[3] Key Laboratory of Acidified Soil Amelioration and Utilization, Ministry of Agriculture and Rural Affairs; Institute of Soil & Fertilizer and Resources & Environment, Jiangxi Academy of Agricultural Sciences, Nanchang 330200, China.

[4] State Key Laboratory of Soil and Sustainable Agriculture, Changshu National Agro-Ecosystem Observation and Research Station, Institute of Soil Science, Chinese Academy of Sciences, Nanjing 211135, China.

[5] Pioneer Center Land-CRAFT, Department of Agroecology, Aarhus University, Aarhus 8000, Denmark.

[6] Department of Forest Resources, University of Minnesota, St. Paul, MN, USA.

[7] Institute for Global Change Biology, University of Michigan, Ann Arbor, MI, USA.

[8] Soil and Crop Sciences Section, School of Integrative Plant Science, Cornell University, Ithaca, New York, USA.

[9] Institute of Biological and Environmental Sciences, University of Aberdeen, Cruickshank Building, St Macha r Drive, Aberdeen AB24 3UU, UK.

[10] CSIC, Global Ecology Unit CREAF-CSIC-UAB, Barcelona, Spain.

[11] Helmholtz Center Munich, Research Unit Comparative Microbiome Analysis, Neuherberg, Germany.

[#] These authors have contributed equally to this work; [*]Corresponding author: Longlong Xia (llxia@issas.ac.cn);

Supplementary Note

Molecular analyses of *chiA* and *nifH* genes. The procedures of measuring *chiA* and *nifH* genes are as followed. Total soil DNA was extracted from 0.5 g of fresh soil using the FastDNA Spin Kit for soil (MP Biomedicals) following the manufacturer's instructions. DNA quality and concentration were determined using a NanoDrop spectrophotometer (PeqLab, Erlangen, Germany). Quantitative real-time PCR (qPCR) was performed on a 7300 Real-Time PCR System (Applied Biosystems, Darmstadt, Germany) using Power SYBR Green Master Mix. Each reaction contained Master Mix, bovine serum albumin (BSA), template DNA (4–10 ng), and primers at a final concentration of 0.2 μM. The bacterial chitinase gene (*chiA*) was quantified using primers GA1F/GA1R (Yergeau et al., 2007), and the nitrogen-fixation gene (*nifH*) using nifH-F/nifH-R (Rösch et al., 2002). The qPCR program consisted of 95°C for 10 min, followed by 40 cycles of 94°C for 45 s, 55°C for 45 s, and 72°C for 45 s. Melting-curve analysis was performed at 95°C for 15 s, 60°C for 30 s, followed by a gradual increase to 95°C at 0.03°C s^{-1}. PCR inhibition was assessed using serial dilutions of soil DNA extracts, and a 1:100 dilution was selected for subsequent analyses. Standard curves were generated from tenfold serial dilutions of plasmids containing the target gene fragments. Amplification specificity was confirmed by a single melting peak and a single band of the expected size on 2% agarose gels stained with ethidium bromide. PCR efficiency was calculated as $Eff = [10^{(-1/slope)} - 1] \times 100\%$, and gene abundances were expressed as gene copies g^{-1} dry soil. Reference: (1) Rösch C et al., 2002. Biodiversity of denitrifying and dinitrogen-fixing bacteria in an acid forest soil. Applied and Environmental Microbiology, 68, 3818–3829. (2) Yergeau et al., 2007. Functional microarray analysis of nitrogen and carbon cycling genes across an Antarctic latitudinal transect. The ISME Journal, 1, 163–179.

Table S1 Two-year averaged soil temperature and water content and the chamber CO_2 concentration during the experimental period from 2020 to 2022

Treatment[a]	Soil Temperature (℃)	Soil water content (%)	CO_2 concentration (ppm)
CK	9.44±0.16	40.15±2.25	465
D	9.55±0.05	33.30±0.10	465
W	11.90±0.30	26.10±0.30	465
D*W	12.30±0.01	21.80±1.00	465
CO_2	9.25±0.35	43.75±0.45	784
CO_2*D	9.60±0.30	35.80±0.70	784
CO_2*W	12.05±0.05	29.60±0.10	784
CO_2*D*W	12.35±0.05	24.95±2.15	784

Treatment[a]	Soil water content (%) during drought period			
	First drought	Second drought	Third drought	Fourth drought
CK	49.62±0.13	29.10±1.01	48.55±0.40	28.31±0.39
D	33.47±1.23	16.11±0.26	29.48±1.38	16.55±0.50
W	26.77±1.29	19.47±0.91	32.16±0.93	14.06±0.67
D*W	21.26±0.86	15.66±0.39	12.29±0.12	11.06±0.26
CO_2	48.78±0.85	37.03±0.78	51.34±0.17	32.50±0.35
CO_2*D	36.29±1.65	18.64±0.44	36.14±1.30	17.69±0.47
CO_2*W	26.80±1.29	18.88±0.75	20.97±0.97	18.16±0.40
CO_2*D*W	20.70±0.46	16.18±0.89	14.32±0.62	17.03±0.38

[a]D, drought; W, warming; CO_2, elevated CO_2 concentration; mean±SE.

Table S2 Two-year averaged shoot/root ratio and shoot and root N content during the experimental period from 2020 to 2022

Treatment[a]	Shoot/root ratio	Shoot N content (%)	Root N content (%)
CK	1.00±0.14	2.0±0.1	1.6±0.1
D	0.72±0.10	2.0±0.1	1.6±0.1
W	1.18±0.16	2.5±0.3	1.6±0.1
D*W	0.76±0.10	2.6±0.1	1.8±0.1
CO_2	1.03±0.14	1.9±0.1	1.6±0.1
CO_2*D	0.75±0.10	2.0±0.1	1.5±0.1
CO_2*W	1.37±0.19	2.2±0.1	1.5±0.1
CO_2*D*W	0.88±0.12	1.9±0.1	1.7±0.1

[a]D, drought; W, warming; CO_2, elevated CO_2 concentration; mean±SE (n=6).

Table S3 Effects of single climate change factor on grass total biomass and shoot/root ratio during the experimental period from 2020 to 2022

Climate change effects[a]		Total biomass (t ha^{-1} yr^{-1})	Shoot/root
Warming	W_0	17.4±0.5a	0.87±0.06a
	W	17.1±0.7a	1.05±0.07a
Drought	D_0	17.0±0.6a	1.15±0.08a
	D	17.5±0.6a	0.78±0.05b
eCO_2	aCO_2	15.1±0.6b	0.91±0.06a
	eCO_2	19.5±0.6a	1.01±0.07a

[a]W_0, ambient temperature; W, warming; D_0, ambient rainfall; D, drought; aCO_2, ambient CO_2 concentration; eCO_2, elevated CO_2 concentration. Values are two-year averaged, which are recalculated across all levels of the other treatments to assess the effects of single climate change factor. Mean±SE.

Table S4 Interactive effects of climate change factor on shoot biomass, N content and shoot N uptake during the experimental period from 2020 to 2022

Effects[a]	D.f.	Shoot biomass		Shoot N content		Shoot N uptake		N uptake from soil	
		F	*P>F*	*F*	*P>F*	*F*	*P>F*	*F*	*P>F*
CO_2	1	62.6	0.0000	29.7	0.0000	13.5	0.0004	13.5	0.05
W	1	3.6	0.06	49.7	0.0000	17.2	0.00008	17.2	0.005
D	1	20.3	0.00002	0.6	0.42	17.3	0.00008	17.3	0.06
CO_2*W	1	0.01	0.92	19.7	0.0000	2.8	0.099	2.8	0.64
CO_2*D	1	0.1	0.75	0.9	0.33	0.3	0.57	0.3	0.22
W*D	1	1.3	0.26	3.3	0.07	4.97	0.03	4.97	0.95
CO_2*D*W	1	1.2	0.28	2.4	0.02	5.4	0.02	5.4	0.02

[a]Linear repeated measures models with one-side test are conducted. Year is treated as a random effect and the effect is significant when $P<0.05$.

Table S5 Effects of climate change factor on shoot biomass and N uptake in different years

Climate change effects (%)[a]		Shoot biomass	Shoot N content	Shoot N uptake
Drought	2020-2021	-11.4	+1.1	-11.5
	2021-2022	-16.9	-3.8	-21.6
eCO_2	2020-2021	+33.7	-15.5	+12.8
	2021-2022	+40.4	-9.7	+23.1
Warming	2020-2021	+12.3	+28.0	+35.1
	2021-2022	+6.5	+11.2	+12.0

[a]Values represent the changes due to climate changes compared to control treatment.

Table S6 Changes (%) in ecosystem plant biomass and N pool under different climate change factors

Biomass/N uptake	Effects	Climate change treatment factors			
	**Single factor**	W	D	CO_2	
	Main effects	-1.8	+2.7	+29.3***	
Total Biomass	**Multiple factors**	W×D	W×CO_2	D×CO_2	W×D×CO_2
	Expected effects	+1.9	+38.5	+30.0	+27.6
	Observed effects	+0.9	+27.6***	+32.6***	+25.7**
	**Single factor**	W	D	CO_2	
	Main effects	+7.3	-15.8**	+35.2***	
Shoot biomass	**Multiple factors**	W×D	W×CO_2	D×CO_2	W×D×CO_2
	Expected effects	-0.8	+46.4	+16.5	+21.4
	Observed effects	-9.8	+45.5***	+13.8**	+17.6*
	**Single factor**	W	D	CO_2	
	Main effects	-9.7	+23.8*	+24.0*	
Root biomass	**Multiple factors**	W×D	W×CO_2	D×CO_2	W×D×CO_2
	Expected effects	+4.6	+31.7	+44.6	+33.7
	Observed effects	+11.7	+12.4	+53.1**	+33.9
	**Single factor**	W	D	CO_2	
	Main effects	+25.7**	-15.1*	+22.9**	
Shoot N pool	**Multiple factors**	W×D	W×CO_2	D×CO_2	W×D×CO_2
	Expected effects	+11.3	+69.1	+3.1	+31.2
	Observed effects	+7.0	+57.0***	+4.3	+22.8
	**Single factor**	W	D	CO_2	
	Main effects	-2.0	+28.9*	+13.6	
Root N pool	**Multiple factors**	W×D	W×CO_2	D×CO_2	W×D×CO_2
	Expected effects	+1.6	+40.2	+45.6	+50.7
	Observed effects	+24.9	+12.1	+46.7*	+45.0

Note: The values are two-year averaged from 2020 to 2022 and the expected and observed percent changes that resulted from two-, three-way interactions are shown. Calculations for two- and three-way interactions were done across all levels of the other treatments. Expected effects were the additive effects sizes of each treatment when imposed individually. The interactive effects tend to be synergistic when the observed effects are greater than the expected effects, otherwise the effects are antagonistic. W, warming; D, drought; CO_2, elevated CO_2 concentration. The significance levels shown for the observed effects indicate differences relative to ambient controls (CK), rather than to the expected effects. *0.01≤*P*<0.05, **0.001≤*P*<0.01, ***P*<0.001. Changes without labeling "*" denote insignificant effects compared with CK. One-way ANOVA followed by a two-sided Tukey HSD test was performed.

Table S7 Changes (%) in plant N uptake and ecosystem N balance under climate change factors

**N fluxes**	**Effects**[a]	**Climate change treatment factors**			
	**Single factor**	W	D	CO_2	
	Main effects	+12.6*	+2.1	+18.7***	
Total N pool	**Multiple factors**	W×D	W×CO_2	D×CO_2	W×D×CO_2
	Expected effects	+7.0	+55.2	+19.9	+39.6
	Observed effects	+14.9	+35.5***	+21.1***	+32.3**
	**Single factor**	W	D	CO_2	
	Main effects	+8.2	-10.5*	-1.9	
Fertilizer N uptake	**Multiple factors**	W×D	W×CO_2	D×CO_2	W×D×CO_2
	Expected effects	-1.3	+6.2	-29.4	-7.5
	Observed effects	-3.2	+6.2	-11.6	+0.4
	**Single factor**	W	D	CO_2	
	Main effects	+13.4*	+4.4	+22.6***	
Soil N uptake	**Multiple factors**	W×D	W×CO_2	D×CO_2	W×D×CO_2
	Expected effects	+8.6	+65.6	+30.7	+50.1
	Observed effects	+18.3*	+41.7***	+28.2**	+39.5**
	**Single factor**	W	D	CO_2	
	Main effects	-3.9	-4.7	-0.2	
Soil N retention	**Multiple factors**	W×D	W×CO_2	D×CO_2	W×D×CO_2
	Expected effects	-4.3	+1.0	-12.2	-9.2
	Observed effects	-8.4	-4.2	-4.8	-7.9
	**Single factor**	W	D	CO_2	
	Main effects	+86.6**	-40.5*	+82.6**	
N balance	**Multiple factors**	W×D	W×CO_2	D×CO_2	W×D×CO_2
	Expected effects	+50.1	+578.2	+56.2	+212.7
	Observed effects	+12.4	+413.9**	+9.0	+56.8
	**Single factor**	W	D	CO_2	
	Main effects	+106.1***	-12.5	+44.6*	
chiA gene	**Multiple factors**	W×D	W×CO_2	D×CO_2	W×D×CO_2
	Expected effects	+1.4	+281.6	+12.4	+59.9
	Observed effects	+65.6**	+222.4***	+25.3	+143.6**

[a]*0.01≤P<0.05, **0.001≤P<0.01, **P<0.001. W, warming; D, drought; CO_2, elevated CO_2 concentration. The significance levels shown for the observed effects indicate differences relative to CK, rather than to the expected effects. Changes without labeling “*” denote insignificant effects compared with CK. One-way ANOVA followed by a two-sided Tukey HSD test was performed.

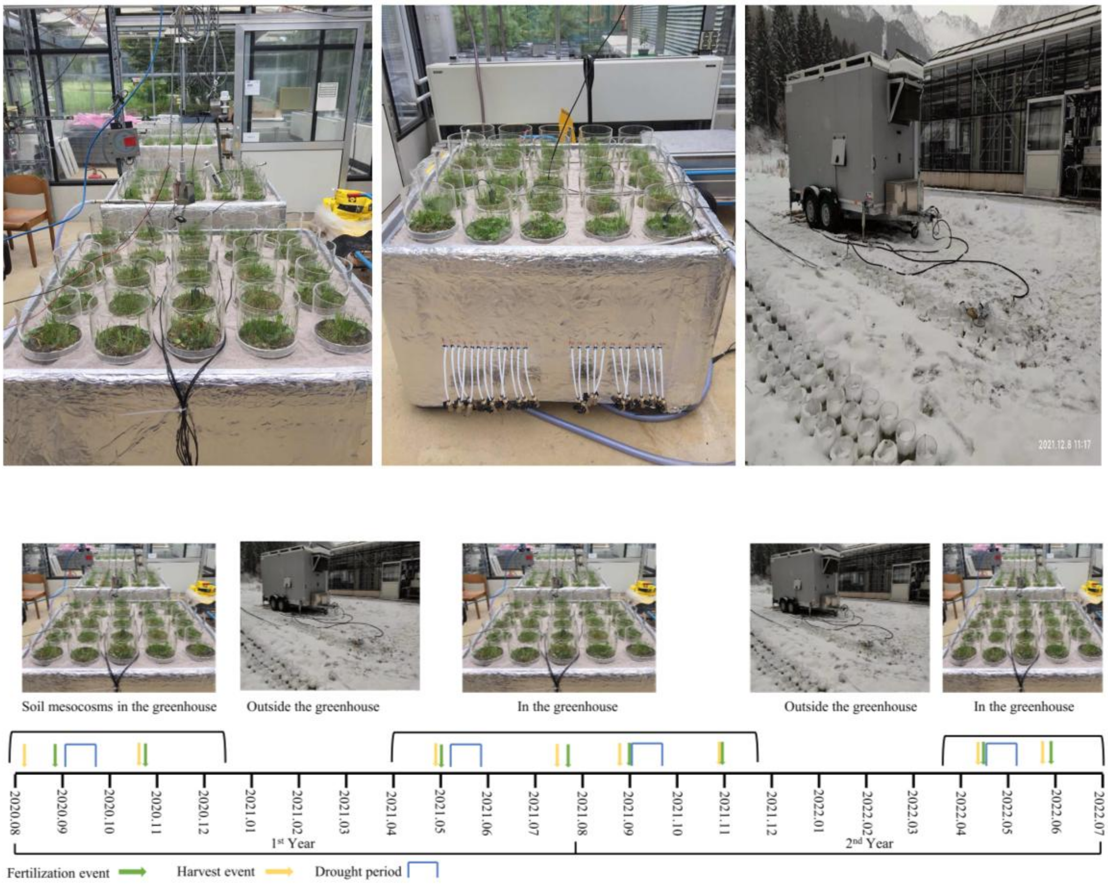

Figure S1. Experimental design of the manipulation of climate changes using intact soil cores and timeline of various agricultural management practices. All soil cores are under manipulated climate change effects during grass-growth season from May to October, which are buried into soil during the non-growth season from November to April.

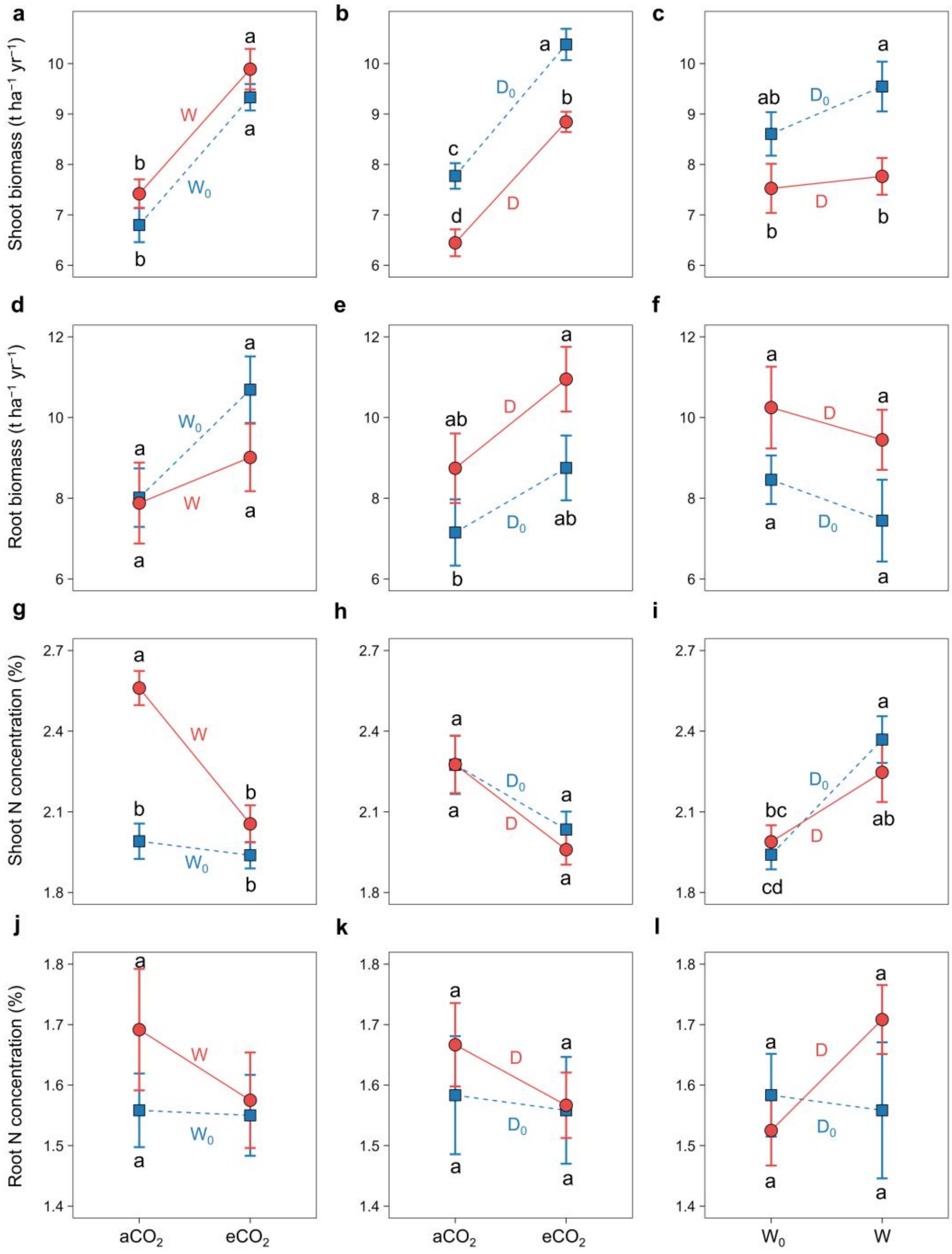

Figure S2. Plant biomass (a−f) and its N concentration (g−l) in relation to pairs of climate change factors. Values are two-year averaged from 2020 to 2022. W_0, ambient temperature; W, warming; D_0, ambient rainfall; D, drought; aCO_2, ambient CO_2 concentration; eCO_2, elevated CO_2 concentration. Values are recalculated across all levels of the other treatments to assess the effects of combined climate change factors. Error bar denotes means ± SE (n=12, biological replicates). Significant differences between groups were determined using one-way ANOVA followed by a two-sided Tukey HSD test. Different letters (e.g., a, b, c) denote statistically significant differences between group means at $P < 0.05$. The exact P values of statistical tests are provided in the Source Data file.

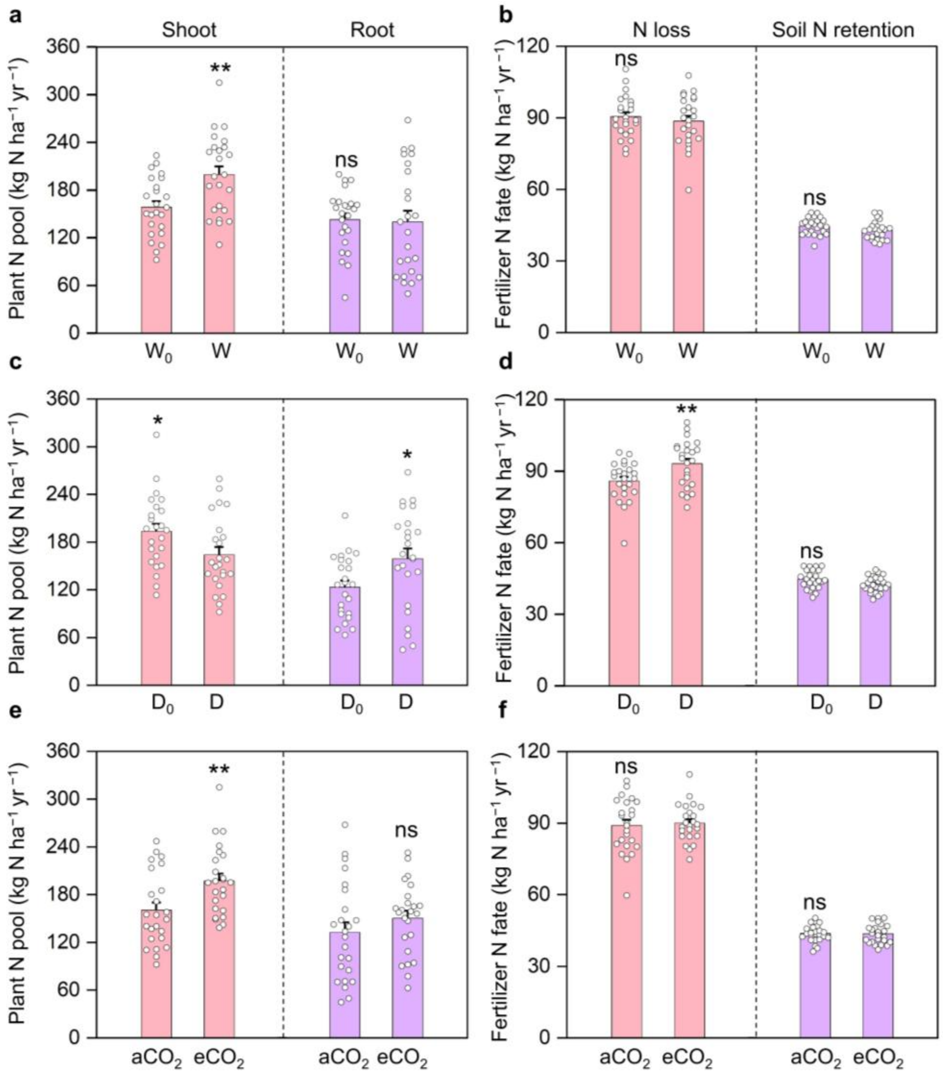

Figure S3. Response of plant N uptake (a, c, e) and fertilizer N fate (b, d, f) to warming, drought and eCO_2. Values are two-year averaged from 2020 to 2022. Effects of warming, drought and eCO_2 on shoot and root N pool (**a**, **c**, **e**), and fertilizer N losses and soil N retention (**b**, **d**, **f**). W_0, ambient temperature; W, warming; D_0, ambient rainfall; D, drought; aCO_2, ambient CO_2 concentration; eCO_2, elevated CO_2 concentration. Values are recalculated across all levels of the other treatments to assess the effects of single climate change factor. Error bar denotes means ± SE (n=24, biological replicates). Significant differences between groups were determined using one-way ANOVA followed by a two-sided Tukey HSD test ($^{*}0.01 \le P < 0.05$, $^{**}0.001 \le P < 0.01$, $^{***}P < 0.001$, $^{ns}0.05 \le P$). The exact *P* values of statistical tests are provided in the Source Data file.

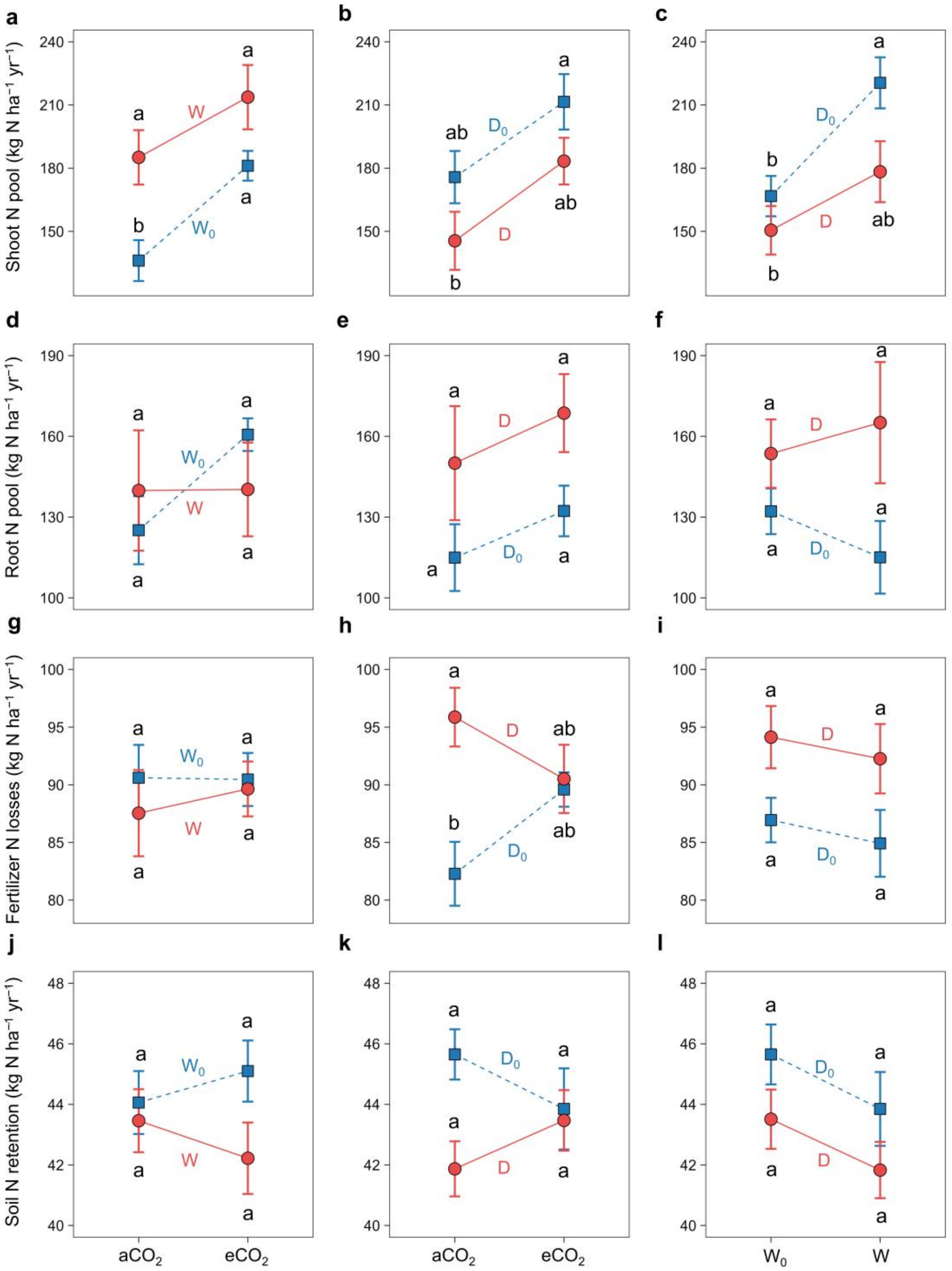

Figure S4. Response of plant N pool (a−f) and fertilizer N fate (g−l) to warming, drought and eCO_2. Values are two-year averaged from 2020 to 2022. W_0, ambient temperature; W, warming; D_0, ambient rainfall; D, drought; aCO_2, ambient CO_2 concentration; eCO_2, elevated CO_2 concentration. Values are recalculated across all levels of the other treatments to assess the effects of combined climate change factors. Error bar denotes means ± SE (n=12, biological replicates). Significant differences between groups were determined using one-way ANOVA followed by a two-sided Tukey HSD test. Different letters (e.g., a, b, c) denote statistically significant differences between group means at $P < 0.05$. The exact P values of statistical tests are provided in the Source Data file.

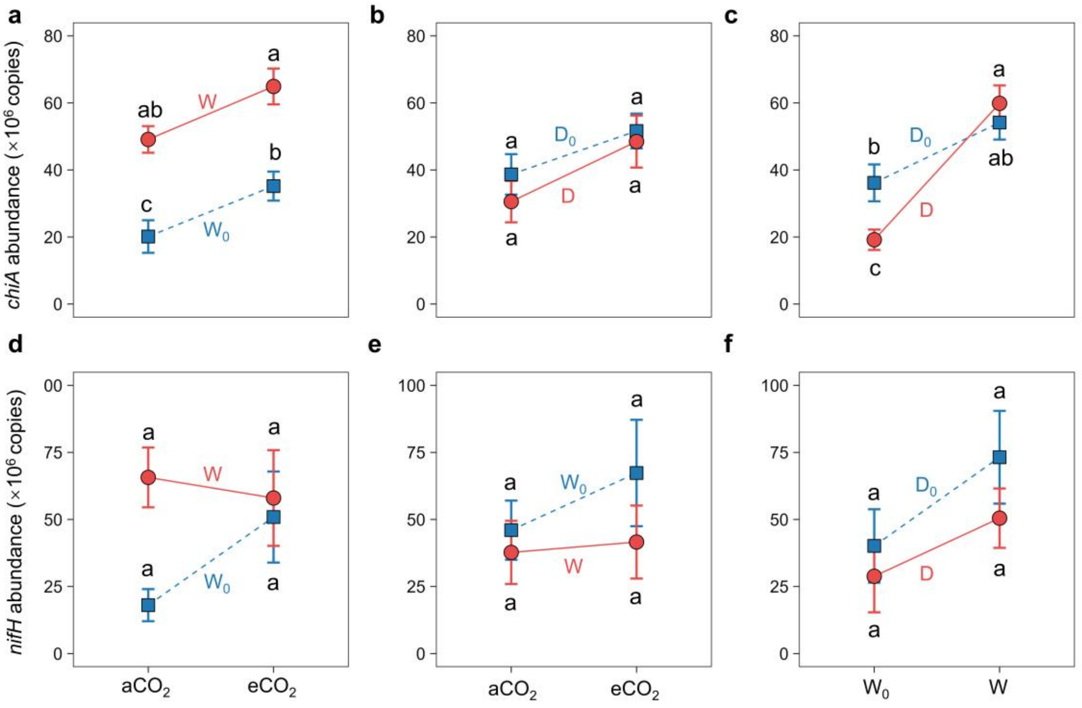

Figure S5. Response of *chiA* (a−c) and *nifH* gene abundance (d−f) to warming, drought and eCO_2. W_0, ambient temperature; W, warming; D_0, ambient rainfall; D, drought; aCO_2, ambient CO_2 concentration; eCO_2, elevated CO_2 concentration. Values are recalculated across all levels of the other treatments to assess the effects of combined climate change factors. Error bar denotes means ± SE (n=12, biological replicates). Significant differences between groups were determined using one-way ANOVA followed by a two-sided Tukey HSD test. Different letters denote statistically significant differences between group ($P < 0.05$).
