# Supplementary material for: Elevated CO2 and warming intensify plant reliance on soil nitrogen reserves despite intensive fertilization
Source: Nat Commun. 2026 Jul 8;17:5979. doi: 10.1038/s41467-026-75147-w (PMC13346951; doi:10.1038/s41467-026-75147-w)
Supplement: Supplementary file 3 — Reporting Summary [file 41467_2026_75147_MOESM3_ESM.pdf]

nature portfolio

Corresponding author(s): Longlong Xia

Last updated by author(s): Jun 11, 2026

Reporting Summary

Nature Portfolio wishes to improve the reproducibility of the work that we publish. This form provides structure for consistency and transparency in reporting. For further information on Nature Portfolio policies, see our Editorial Policies and the Editorial Policy Checklist.

Statistics

For all statistical analyses, confirm that the following items are present in the figure legend, table legend, main text, or Methods section.

n/a	Confirmed	
☐	☒	The exact sample size (*n*) for each experimental group/condition, given as a discrete number and unit of measurement
☐	☒	A statement on whether measurements were taken from distinct samples or whether the same sample was measured repeatedly
☐	☒	The statistical test(s) used AND whether they are one- or two-sided<br>*Only common tests should be described solely by name; describe more complex techniques in the Methods section.*
☒	☐	A description of all covariates tested
☐	☒	A description of any assumptions or corrections, such as tests of normality and adjustment for multiple comparisons
☐	☒	A full description of the statistical parameters including central tendency (e.g. means) or other basic estimates (e.g. regression coefficient) AND variation (e.g. standard deviation) or associated estimates of uncertainty (e.g. confidence intervals)
☐	☒	For null hypothesis testing, the test statistic (e.g. *F*, *t*, *r*) with confidence intervals, effect sizes, degrees of freedom and *P* value noted<br>*Give P values as exact values whenever suitable.*
☒	☐	For Bayesian analysis, information on the choice of priors and Markov chain Monte Carlo settings
☒	☐	For hierarchical and complex designs, identification of the appropriate level for tests and full reporting of outcomes
☒	☐	Estimates of effect sizes (e.g. Cohen's *d*, Pearson's *r*), indicating how they were calculated

Our web collection on statistics for biologists contains articles on many of the points above.

Software and code

Policy information about availability of computer code

Data collection	This study does not involve this aspect.
Data analysis	Significant differences between groups were determined using one-way ANOVA followed by a two-sided Tukey HSD test. Interaction effects among climate change factors were quantified using a repeated measures mixed model with both fixed and random effects that deploys a maximum likelihood approach using RStudio (3.4.3).

For manuscripts utilizing custom algorithms or software that are central to the research but not yet described in published literature, software must be made available to editors and reviewers. We strongly encourage code deposition in a community repository (e.g. GitHub). See the Nature Portfolio guidelines for submitting code & software for further information.

Data

Policy information about availability of data

All manuscripts must include a data availability statement. This statement should provide the following information, where applicable:

- Accession codes, unique identifiers, or web links for publicly available datasets
- A description of any restrictions on data availability
- For clinical datasets or third party data, please ensure that the statement adheres to our policy

The data generated in this study are provided in the Source Data file and Supplementary Information.

Research involving human participants, their data, or biological material

Policy information about studies with human participants or human data. See also policy information about sex, gender (identity/presentation), and sexual orientation and race, ethnicity and racism.

Reporting on sex and gender	This study does not involve this aspect.
Reporting on race, ethnicity, or other socially relevant groupings	This study does not involve this aspect.
Population characteristics	This study does not involve this aspect.
Recruitment	This study does not involve this aspect.
Ethics oversight	This study does not involve this aspect.

Note that full information on the approval of the study protocol must also be provided in the manuscript.

Field-specific reporting

Please select the one below that is the best fit for your research. If you are not sure, read the appropriate sections before making your selection.

☐ Life sciences ☐ Behavioural & social sciences ☒ Ecological, evolutionary & environmental sciences

For a reference copy of the document with all sections, see nature.com/documents/nr-reporting-summary-flat.pdf

Ecological, evolutionary & environmental sciences study design

All studies must disclose on these points even when the disclosure is negative.

Study description	Understanding how ecosystems sustain plant nitrogen (N) supply under climate change is critical for predicting future productivity, yet it remains unclear whether the response of plant N demand to climate change is met by external N inputs or mobilization of soil organic N (SON) reserves. Here, we evaluate how climate change alters ecosystem N sourcing, using a two-year multifactor experiment combining elevated CO2 (eCO2), warming, and drought, with in-situ 15N tracing in intact plant-soil mesocosms from a pre-Alpine grassland. Contrary to prevailing expectations that fertilization buffers ecosystems against N limitation, we showed that climate change consistently increased plant reliance on soil-derived N despite intensive fertilizer inputs. Across all treatments, plants obtained the majority of their N from soil (82–88%), acquiring 4.6–7.3 times more N from SON than from fertilizer despite high N inputs (180 kg N ha⁻1 yr⁻1). Warming and eCO2 consistently increased plant uptake of soil-derived N while leaving fertilizer-derived N acquisition largely unchanged. As a result, plant N export exceeded fertilizer inputs, driving persistent ecosystem N deficits and progressive depletion of soil N stocks. Moreover, multifactor climate treatments amplified this effect, with the strongest soil N mining occurring under combined warming and elevated CO2. These findings reveal a fundamental reorganization of ecosystem N cycling, whereby climate change drives biological mining of soil N to sustain plant growth. This climate-driven soil N mining threatens long-term sustainability of N-rich agroecosystems and underscores the need to reconsider N management strategies under rapidly changing climate.
Research sample	Grass shoot and root biomass were sampled for analyzing N content and the excess 15N to quantify the proportional contributions (%) of fertilizer-derived and soil-derived N uptake. Soil samples (0-25cm) were collected for measuring 15N retention, as well as the the abundances of the nifH and ChiA genes.
Sampling strategy	After harvesting, plant biomass sample was initially air-dried by turning it every two days for one week, then oven-dried at 60°C to constant weight. Dried material was weighed, finely chopped, homogenized, and a representative subsample was ground using a ball mill at 30rpm for 3 minutes for further analyses. Soil samples (0-25cm) from the mesocosms were composited, homogenized, air-dried, and sieved (<2 mm) to obtain a representative bulk sample for all subsequent treatments and analyses.
Data collection	The N content and excess 15N data of the plant and soil samples were automatically measured using a series of related instruments, which were further recorded and analyzed by the authors.
Timing and spatial scale	The study was conducted over 2 years from 2020.07 to 2022.07.
Data exclusions	No data were excluded from the analyses.
Reproducibility	All attempts to repeat the experiment were successful.
Randomization	A randomized complete block design with twelve replications was used in the climate change manipulation experiment.
Blinding	No blinding was required.

Did the study involve field work? ☒ Yes ☐ No

Field work, collection and transport

Field conditions	We collected a total of 100 undisturbed plant–soil mesocosms (8 treatments × 12 replicates, and 4 held in reserve) using stainless-steel cylinders (17 cm diameter × 25 cm height; open at both ends with a sharpened lower edge) from grasslands adjacent to the IMK-IFU campus (47°28′N, 11°04′E; 720 m above sea level) in Germany, with a mean annual temperature and mean growing season temperature of 8.2°C and 10.6°C, respectively, and annual precipitation of 1,386 mm (mean of 2012–2021). The soils are classified as Calcaric Cambisol, with a top soil (0–15cm) bulk density of 0.35 g cm^{-3}, pH of 6.83 and organic carbon and total N contents of 53.5 g C kg^{-1} and SDW 6.3 g N kg^{-1} SDW, respectively.
Location	Experimental treatments were implemented in the IMK-IFU controlled greenhouse facility (47°28′N, 11°04′E; 720 m above sea level), with a mean annual temperature and mean growing season temperature of 8.2°C and 10.6°C, respectively, and annual precipitation of 1,386mm (mean of 2012–2021).
Access & import/export	The experiments were conducted in the IMK-IFU controlled greenhouse facility. Access to the site was authorized by the institute, and no specific permits were required for soil and plant sample collection. All biological samples were processed and analyzed domestically within Germany, and no international export of physical samples or genetic resources was involved.
Disturbance	No disturbance to wildlife or natural habitats occurred due to the experiment.

Reporting for specific materials, systems and methods

We require information from authors about some types of materials, experimental systems and methods used in many studies. Here, indicate whether each material, system or method listed is relevant to your study. If you are not sure if a list item applies to your research, read the appropriate section before selecting a response.

Materials & experimental systems

n/a	Involved in the study	
☒	☐	Antibodies
☒	☐	Eukaryotic cell lines
☒	☐	Palaeontology and archaeology
☒	☐	Animals and other organisms
☒	☐	Clinical data
☒	☐	Dual use research of concern
☐	☒	Plants

Methods

n/a	Involved in the study	
☒	☐	ChIP-seq
☒	☐	Flow cytometry
☒	☐	MRI-based neuroimaging

Dual use research of concern

Policy information about dual use research of concern

Hazards

Could the accidental, deliberate or reckless misuse of agents or technologies generated in the work, or the application of information presented in the manuscript, pose a threat to:

No	Yes	
☒	☐	Public health
☒	☐	National security
☒	☐	Crops and/or livestock
☒	☐	Ecosystems
☒	☐	Any other significant area

Experiments of concern

Does the work involve any of these experiments of concern:

No	Yes	
☒	☐	Demonstrate how to render a vaccine ineffective
☒	☐	Confer resistance to therapeutically useful antibiotics or antiviral agents
☒	☐	Enhance the virulence of a pathogen or render a nonpathogen virulent
☒	☐	Increase transmissibility of a pathogen
☒	☐	Alter the host range of a pathogen
☒	☐	Enable evasion of diagnostic/detection modalities
☒	☐	Enable the weaponization of a biological agent or toxin
☒	☐	Any other potentially harmful combination of experiments and agents

Plants

Seed stocks	This study does not involve this aspect.
Novel plant genotypes	This study does not involve this aspect.
Authentication	This study does not involve this aspect.
