# Supplementary material for: Elevated CO2 and warming intensify plant reliance on soil nitrogen reserves despite intensive fertilization
Source: Nat Commun. 2026 Jul 8;17:5979. doi: 10.1038/s41467-026-75147-w (PMC13346951; doi:10.1038/s41467-026-75147-w)
Supplement: Supplementary file 1 — Peer Review file [file 41467_2026_75147_MOESM1_ESM.pdf]

nature portfolio

Peer Review File

Elevated CO2 and warming intensify plant reliance on soil nitrogen reserves despite intensive fertilization

Corresponding Author: Professor Longlong Xia

This file contains all reviewer reports in order by version, followed by all author rebuttals in order by version.

Version 0:

Reviewer comments:

Reviewer #1

(Remarks to the Author)
The manuscript by Zhu et al. addresses a fundamental and unresolved question in global change ecology: whether the intensified plant N demand under climate change will be met through external inputs or through increased mobilization of internal soil organic nitrogen reserves. By integrating advanced isotopic source-partitioning approaches with a well-designed full-factorial climate manipulation experiment (elevated CO2, warming, and drought), the authors demonstrate that, contrary to the prevailing assumption that high fertilizer inputs buffer ecosystems against N limitation, plants consistently derived 84–89% of their N from SON across all treatments. Furthermore, warming and elevated CO2 increased soil-derived N uptake by 20–25% without affecting fertilizer-derived N uptake, resulting in persistent ecosystem N deficits and net soil N depletion (–3% to –18%). These findings substantially reshape our understanding of ecosystem N cycling under climate change, introducing the concept of climate-driven soil N mining and raising important concerns regarding the long-term sustainability of current N management strategies.
The use of in situ 15N tracing across eight treatment combinations, including full interaction effects, represents a major methodological strength and remains rare in the literature. To the best of my knowledge, this study provides the most comprehensive and sophisticated assessments to date of the combined effects of elevated CO2, warming, and drought on plant–soil N cycling using advanced isotopic tracing. The implementation of multiple 15N labeling events within a fully factorial and well-maintained experimental framework is both technically demanding and resource-intensive, underscoring the exceptional value of the resulting dataset for both empirical and modeling studies.
Overall, the manuscript is highly original and makes a significant contribution to the field, offering a novel perspective on nitrogen cycling in intensively fertilized ecosystems, with important implications for N management under a changing climate. The manuscript is clearly written, the data analyses are appropriate, and the results are robust. The discussion is well developed, effectively linking mechanistic insights (e.g., ChiA gene abundance, biomass allocation) to ecosystem-level responses (e.g., total 15N recovery, N balance and soil N depletion). I enthusiastically recommend its publication in Nature Communications after minor revisions aimed at improving methodological clarity and strengthening the alignment between the data presented and the conclusions drawn.
1. I appreciate the full-factorial climate manipulation design of the study, which uses +300 ppm CO2 (to ~784 ppm), +2.5°C warming, and a 30% reduction in growing-season precipitation. While these values are within the range of future climate projections, the authors should provide a clearer justification for why these specific levels were selected for the experiment based on IPCC SSP scenarios.
2. Abstract: Line 33–34: The phrase “whether the response of plant N demand … is met by external N inputs or mobilization of SON reserves” is clear and effective. I would suggest to introduce SON biological mining here, as it is a central concept introduced later (e.g., “mobilization of SON reserves, termed as biological soil N mining”).
3. Introduction: Line 80–86: The introduction of the “biological mining of soil N” concept is well placed. However, the transition from previous work to the current knowledge gap could be sharpened. Specifically, state explicitly that no previous study has simultaneously quantified fertilizer- vs. soil-derived N uptake under combined eCO2, warming, and drought in a single experiment. This would reinforce novelty.
4. Results: Lines 132–134: More data information are needed for the results about “with effects exceeding those of warming or drought alone”. Current version is not clear.
5. Lines 154–155, 166–167, specifically demonstrate whether these N dynamic changes under climate changes are significant or not by adding P values.
6. Discussion: Lines 249–253: The explanation for reduced NUE under climate change (i.e., slurry dehydration and crust formation restricting microbial breakdown) is plausible but appears to be based on visual observation. Please clarify this as

a proposed mechanism or soften the wording (e.g., “may have dehydrated and formed a crust, potentially restricting microbial access”).
7. Lines 256–258: The increase in ChiA gene abundance (10–146%) provides compelling evidence for enhanced microbial decomposition. It would be helpful to briefly acknowledge that gene abundance does not necessarily equate to activity, while noting that the consistency with N uptake patterns strengthens the inference.
8. Lines 286–302: The recommendations (e.g., slurry incorporation, nitrification inhibitors, reduced cutting frequency) are reasonable and supported by literature. However, please acknowledge more explicitly that these strategies were not directly tested under the climate treatments in this study. A brief statement noting the need for validation under future climate conditions would strengthen this section.
9. Methodology: Biological N fixation (BNF) is estimated at 15 kg N ha-1 yr-1 based on 3% legume abundance and assumed constant across treatments because nifH gene abundance did not change (Fig. 3h). Please state this explicitly in the Methods (and consider moving it earlier for clarity): “Because nifH gene abundance did not differ significantly among treatments (Fig. 3h), BNF was assumed unaffected by climate manipulation.”
10. The labeling uncertainty is appropriately acknowledged. To further reassure readers, please provide a quantitative estimate of potential bias based on labeling efficiency (>90%), e.g., “the potential bias is estimated to be <5%.”
Other minor comments:
1. Lines 116–117: “persistent negative ecosystem N balances” – the word “persistent” implies multi-year directionality. Given the two-year study, consider “sustained” or “repeated” unless longer-term data are available.
2. Figure 1 caption: The significance notation includes “ ＊ ＊ 0.05<P at the end. This appears to be a typo – should it be “ns” or “P > 0.05”? Please correct.
3. Supplementary Tables 6 and 7: Authors need to clearly demonstrate that if the changes are not labelled with*, these changes are not significantly with ambient controls.

Reviewer #2

(Remarks to the Author)
General comments
The authors investigated how climate change affects the nitrogen (N) sources of plant N uptake using a multifactor experiment. Based on a 15N tracing approach, they found that soil-derived N, rather than fertilizer N, is the main N source sustaining plant N demand. They further attempted to predict the ecosystem N balance. However, I have the following concerns regarding these results.
1. The author added 15NH4+ and urea-15N to cattle slurry. However, they did not report the N forms present in the cattle slurry and their individual proportions. Dissolved N in cattle slurry includes NH4, urea-N, and other N forms. These unlabeled N forms may serve as potential N sources for plants and could affect the recovery of 15N in plant (leading to an overestimation of the contribution of soil N). In addition, the authors reported that the added fertilizer N (NH4 and urea) labeled approximately 90% of all N forms in the slurry, but the cited reference reported a proportion of 3/4. These issues introduce uncertainties to the results.
2. The calculation of N balance is coarse. The authors estimated BNF based on previous empirical relationships derived from comparable grasslands. However, the studied montane grassland is intensive managed (with cattle slurry). This difference may bias the estimation of BNF.
3. Cattle slurry addition introduces both C and N into the soil, thereby affecting both C and N availability. C availability may further influence microbial activity and its response to climate change. The current discussion does not mention the effect of C. The introduction also largely ignores the role of C addition. Therefore, the current results may reflect the specific conditions of organic fertilizer application rather than broader fertilization scenarios.
Minor comments
Line 368, “0.2 g N kg-1 FW organic N” Does this organic N refer to unextractable organic N? Please specify.
Line 368-369, Please report the results of N forms and their individual contents (e.g., unextractable N, dissolved N (NH4, urea-N, other dissolved N)). What is the proportion of urea in total dissolved N? I checked references [17] and [22] and did not find these results.
Line 373, “label around 90% of all N forms in the slurry” A previous study reported “label around 3/4 of all N forms in the slurry.” In addition, why did you cite reference [38] here?
Line 434, labeling efficiency? Please clarify.

Open Access This Peer Review File is licensed under a Creative Commons Attribution 4.0 International License, which permits use, sharing, adaptation, distribution and reproduction in any medium or format, as long as you give appropriate credit to the original author(s) and the source, provide a link to the Creative Commons license, and indicate if changes were made.
In cases where reviewers are anonymous, credit should be given to 'Anonymous Referee' and the source.
The images or other third party material in this Peer Review File are included in the article's Creative Commons license, unless indicated otherwise in a credit line to the material. If material is not included in the article's Creative Commons license and your intended use is not permitted by statutory regulation or exceeds the permitted use, you will need to obtain permission directly from the copyright holder.
To view a copy of this license, visit https://creativecommons.org/licenses/by/4.0/

Dear reviewers,

On behalf of my co-authors, thank you very much for giving us the opportunity to revise our manuscript "**Elevated CO_2 and warming intensify plant reliance on soil nitrogen reserves despite intensive fertilization**" [NCOMMS-26-032621]. We very much appreciated the insightful and constructive feedback from the reviewers. In response, we have carefully examined each comment and undertaken a detailed revision of our manuscript and the revised contents are highlighted by blue color. Please find our point-by-point responses to reviewers' comments below:

Reviewer Comments:

Reviewer #1:

General Comments:

The manuscript by Zhu et al. addresses a fundamental and unresolved question in global change ecology: whether the intensified plant N demand under climate change will be met through external inputs or through increased mobilization of internal soil organic nitrogen reserves. By integrating advanced isotopic source-partitioning approaches with a well-designed full-factorial climate manipulation experiment (elevated CO_2, warming, and drought), the authors demonstrate that, contrary to the prevailing assumption that high fertilizer inputs buffer ecosystems against N limitation, plants consistently derived 84–89% of their N from SON across all treatments. Furthermore, warming and elevated CO_2 increased soil-derived N uptake by 20–25% without affecting fertilizer-derived N uptake, resulting in persistent ecosystem N deficits and net soil N depletion (–3% to –18%). These findings substantially reshape our understanding of ecosystem N cycling under climate change, introducing the concept of climate-driven soil N mining and raising important concerns regarding the long-term sustainability of current N management strategies.

The use of in situ ^{15}N tracing across eight treatment combinations, including full interaction effects, represents a major methodological strength and remains rare in the literature. To the best of my knowledge, this study provides the most comprehensive and sophisticated assessments to date of the combined effects of elevated CO_2, warming, and drought on plant–soil N cycling using advanced isotopic tracing. The implementation of multiple ^{15}N labeling events within a fully factorial and well-maintained experimental framework is both technically demanding and resource-intensive, underscoring the exceptional value of the resulting dataset for both empirical and modeling studies.

Overall, the manuscript is highly original and makes a significant contribution to the field, offering a novel perspective on nitrogen cycling in intensively fertilized ecosystems, with important implications for N management under a changing climate. The manuscript is clearly

written, the data analyses are appropriate, and the results are robust. The discussion is well developed, effectively linking mechanistic insights (e.g., ChiA gene abundance, biomass allocation) to ecosystem-level responses (e.g., total ^{15}N recovery, N balance and soil N depletion). I enthusiastically recommend its publication in Nature Communications after minor revisions aimed at improving methodological clarity and strengthening the alignment between the data presented and the conclusions drawn.

Response: Thank you very much for your positive comments on our study. We have carefully addressed all comments as detailed below.

Specific comments:

1. I appreciate the full-factorial climate manipulation design of the study, which uses +300 ppm CO_2 (to ~784 ppm), +2.5°C warming, and a 30% reduction in growing-season precipitation. While these values are within the range of future climate projections, the authors should provide a clearer justification for why these specific levels were selected for the experiment based on IPCC SSP scenarios.

Response: Thanks for your suggestion. Based on your suggestions, we have provided a clearer justification for why these specific levels were selected for the experiment based on IPCC SSP scenarios. The newly added contents read "These levels were selected to represent projected climatic conditions for Central Europe under intermediate- to high-emission scenarios by the late 21st century. The elevated CO_2 treatment approximates concentrations expected under SSP2-4.5 to SSP3-7.0 scenarios, while the +2.5 °C warming reflects regional temperature increases projected for the same range of scenarios. The 30% reduction in growing-season precipitation represents current-to-projected seasonal water deficits and increasing drought frequency in the pre-alpine region" (**Lines 342 – 348**).

2. Abstract: Line 33–34: The phrase "whether the response of plant N demand … is met by external N inputs or mobilization of SON reserves" is clear and effective. I would suggest to introduce SON biological mining here, as it is a central concept introduced later (e.g., "mobilization of SON reserves, termed as biological soil N mining").

Response: Agreed and revised. The new sentence now reads "….yet it remains unclear whether the response of plant N demand to climate change is met by external N inputs or mobilization of soil organic N (SON) reserves (i.e., biological soil N mining)" (**Lines 34 – 36**).

3. Introduction: Line 80–86: The introduction of the "biological mining of soil N" concept is well placed. However, the transition from previous work to the current knowledge gap could be sharpened. Specifically, state explicitly that no previous study has simultaneously quantified

fertilizer- vs. soil-derived N uptake under combined eCO_2, warming, and drought in a single experiment. This would reinforce novelty.

Response: Thanks for your suggestion. Based on your suggestions, we have reinforced the novelty of the study by adding the following sentence into the Introduction "However, no previous study has simultaneously quantified the relative contributions of fertilizer-derived versus soil-derived N to plant uptake under eCO_2, warming and drought within a single experimental framework, contributing to major uncertainties in projections of nitrogen-climate feedbacks in terrestrial ecosystems" **(Lines 79–82)**.

4. Results: Lines 132–134: More data information are needed for the results about "with effects exceeding those of warming or drought alone". Current version is not clear.

Response: Sorry for the confusion. We have revised the sentence to "eCO_2 significantly enhanced both shoot and root biomass by 24–35.2% ($P<0.001$; Fig. 1f), exceeding the effects of warming or drought applied individually" (**Lines 135–137**).

5. Lines 154–155, 166–167, specifically demonstrate whether these N dynamic changes under climate changes are significant or not by adding P values.

Response: We have added P values to these sentences (**Lines 158 and 170**).

6. Discussion: Lines 249–253: The explanation for reduced NUE under climate change (i.e., slurry dehydration and crust formation restricting microbial breakdown) is plausible but appears to be based on visual observation. Please clarify this as a proposed mechanism or soften the wording (e.g., "may have dehydrated and formed a crust, potentially restricting microbial access").

Response: Thanks for your suggestion. Based on your suggestions, we have revised the sentence to "Visual observations during the experiment confirmed that, under warming and drought conditions, the solid fraction may have dehydrated quickly and formed a dry crust, potentially restricting microbial breakdown, making it even more difficult for plants to access N from the crust" for clarification (**Lines 252–255**).

7. Lines 256–258: The increase in ChiA gene abundance (10–146%) provides compelling evidence for enhanced microbial decomposition. It would be helpful to briefly acknowledge that gene abundance does not necessarily equate to activity, while noting that the consistency with N uptake patterns strengthens the inference.

Response: Thanks for your suggestion. We have corrected the sentences into "Enhanced microbial assimilation of fertilizer-derived ammonium (NH_4^+) may induce stoichiometric imbalance and stimulates SON mineralization to satisfy microbial and plant N uptake, consistent with the microbial N pump mechanism. In addition, eCO_2-induced increases in root

exudation, combined with slurry-derived carbon inputs, likely further stimulated microbial decomposition of soil organic matter, intensifying microbial N mining. Although gene abundance does not necessarily directly reflect microbial process rates, the consistent increase in ChiA abundance (10–146%), together with enhanced soil-derived plant N uptake, supports the inference that warming and eCO_2 stimulated microbial decomposition and SON mobilization (Fig. 3)" to make it clearer **(Lines 259–267)**.

8. Lines 286–302: The recommendations (e.g., slurry incorporation, nitrification inhibitors, reduced cutting frequency) are reasonable and supported by literature. However, please acknowledge more explicitly that these strategies were not directly tested under the climate treatments in this study. A brief statement noting the need for validation under future climate conditions would strengthen this section.

Response: Thanks for your valuable and insightful suggestion. We have explicitly acknowledged that these strategies were not directly tested under the climate treatments in this study as well as highlighting the necessity to explore the effectiveness of these management practices in future studies. The revised sentences now read "However, the effectiveness of these strategies under future climate conditions was not directly tested in this study. Given the strong sensitivity of soil N cycling to climate drivers, their effectiveness under future climate change requires further experimental validation. Long-term, integrative studies linking plant N uptake, soil N transformations, and N losses are essential to improve predictive models and guide sustainable management strategies under a rapidly changing climate" **(Lines 312–317)**.

9. Methodology: Biological N fixation (BNF) is estimated at 15 kg N ha^{-1} yr^{-1} based on 3% legume abundance and assumed constant across treatments because nifH gene abundance did not change (Fig. 3h). Please state this explicitly in the Methods (and consider moving it earlier for clarity): "Because nifH gene abundance did not differ significantly among treatments (Fig. 3h), BNF was assumed unaffected by climate manipulation."

Response: Thanks for your suggestion. We have reorganized the sequence of the sentences and added discussion about the uncertainty associated with the estimation of BNF in this study. The revised contents read "As the abundance of the nifH gene did not significantly differ among treatments (Fig. 3; Supplementary Fig. 5), BNF was assumed to be unaffected by climate manipulation. BNF was estimated using observed legume abundance (3% of total biomass) and established empirical relationships from comparable grasslands, yielding an average BNF rate of 15 kg N ha^{-1} yr^{-1}. We acknowledge that this approach may introduce uncertainty because the studied grassland was intensively managed with slurry application. However, legumes constituted only a small fraction of total biomass, and BNF contributed minimally to total ecosystem N inputs relative to slurry fertilization" **(Lines 492–499)**.

10. The labeling uncertainty is appropriately acknowledged. To further reassure readers, please provide a quantitative estimate of potential bias based on labeling efficiency, e.g., "the potential bias is estimated to be <5%."

Response: Thanks for your suggestions. The ^{15}N labelling approach used in this study effectively labelled the majority (>85%) of plant-available N forms in the slurry. Because a small fraction of slurry organic N was not uniformly labelled, recovery-based estimates of fertilizer-derived N may be slightly underestimated, with a corresponding slight overestimation of soil-derived N. Based on the proportion of unlabeled N and tracer enrichment levels, the potential bias is estimated to be minimal (<5%). Importantly, fertilizer N recovery calculations were based on the actual ^{15}N input, ensuring that this minor uncertainty does not affect the central conclusion that plant N uptake is dominated by soil-derived sources under climate change. We have added a paragraph in the revised manuscript to explicitly acknowledge and quantify this uncertainty.

The added paragraph now read "Several uncertainties associated with our experimental approach should be acknowledged. First, the ^{15}N-labeling strategy primarily targeted the dominant readily plant-available N forms in the slurry (NH_4^+-N and urea-N), which together accounted for the vast majority of total slurry N (see Methods). A small fraction of particulate organic N remained unlabeled and may have been partially taken up by plants without being traced in the fertilizer-derived N pool. As a result, estimates of fertilizer N recovery likely represent a conservative bound, with a minor (<5%) potential bias toward underestimating fertilizer-derived uptake and overestimating soil-derived N. However, this is unlikely to affect our central conclusion that plant N uptake was overwhelmingly dominated by soil-derived N across treatments. Second, biological N fixation was estimated using empirical relationships and assumed to be invariant among treatments, consistent with the absence of significant differences in nifH gene abundance under climate manipulation. Although this introduces uncertainty into ecosystem N budgets, BNF contributed only a minor fraction of total N inputs relative to slurry fertilization. Finally, because the experiment focused on slurry-based organic fertilization, observed responses may partly reflect interactions between climate change and slurry-derived carbon inputs. Future work should test whether similar patterns of climate-driven soil N mining occur under mineral fertilizer systems. Nevertheless, these uncertainties do not alter the main conclusion that climate change intensified plant reliance on soil-derived N despite intensive organic N fertilization" **(Lines 318–335)**.

Minor comments:

11. Lines 116–117: "persistent negative ecosystem N balances" – the word "persistent" implies multi-year directionality. Given the two-year study, consider "sustained" or "repeated" unless longer-term data are available.

Response: Thanks for your suggestion. We have changed "persistent" to "sustained" **(Line 119)**.

12. Figure 1 caption: The significance notation includes "∗∗0.05 0.05"? Please correct.

Response: Sorry for the confusion. We have changed the expression to "*$0.01 \le P < 0.05$, **$0.001 \le P < 0.01$, ***$P < 0.001$, $^{ns}0.05 \le P$" **(see Figure captions)**.

13. Supplementary Tables 6 and 7: Authors need to clearly demonstrate that if the changes are not labelled with*, these changes are not significantly with ambient controls.

Response: Agreed and corrected **(see Supplementary Tables 6 and 7 captions)**.

Reviewer #2:

Specific comments:

1. The author added $^{15}NH_4^+$ and urea-^{15}N to cattle slurry. However, they did not report the N forms present in the cattle slurry and their individual proportions. Dissolved N in cattle slurry includes NH_4, urea-N, and other N forms. These unlabeled N forms may serve as potential N sources for plants and could affect the recovery of ^{15}N in plant (leading to an overestimation of the contribution of soil N). In addition, the authors reported that the added fertilizer N (NH_4 and urea) labeled approximately 90% of all N forms in the slurry, but the cited reference reported a proportion of 3/4. These issues introduce uncertainties to the results.

Response: Thanks very much for your constructive comments and suggestions. Following your suggestion, we have now reported the major N forms and their proportions in the cattle slurry in the Methods section: "On average, the slurry had a pH of 7.5 and a total nitrogen content of 2.0 g N kg^{-1} fresh weight (FW), comprising 0.2 g N kg^{-1} FW unextractable organic N, and 1.8 g N kg^{-1} FW dissolved N, including ammonium (NH_4^+-N) and urea-N" **(Lines 406–408)**. The efficiency of the ^{15}N-labelling approach depends on the relative abundance of dissolved NH_4^+-N and urea-N in the slurry, which typically accounts for ~80–90% of total slurry N. The previously cited study used slurry obtained from different farms that contained a larger proportion of solid unextractable organic N and lower dissolved N content, resulting in a lower labelling efficiency (~75%). In contrast, the slurry used in our study was dominated by dissolved NH_4^+-N and urea-N, allowing the ^{15}N tracer approach to effectively label the majority (>85%, approximately 90%) of plant-available N forms.

We agree that a small fraction of unlabeled organic N could potentially contribute to plant uptake and therefore lead to slight underestimation of fertilizer-derived N and corresponding overestimation of soil-derived N. However, based on the proportion of unlabeled N and tracer enrichment levels, the potential bias is estimated to be minimal (<5%). Importantly, fertilizer N recovery calculations were based on the actual ^{15}N input, ensuring that this uncertainty does

not affect the central conclusion that plant N uptake was predominantly sustained by soil-derived N under climate change (**Lines 419–426**). To avoid confusion, we have removed the previous citation reporting lower labelling efficiency and have added a dedicated paragraph in the revised manuscript explicitly acknowledging and quantifying the uncertainties associated with the ^{15}N-labelling approach (**Lines 318–335**).

The added paragraph now reads "Several uncertainties associated with our experimental approach should be acknowledged. First, the ^{15}N-labeling strategy primarily targeted the dominant readily plant-available N forms in the slurry (NH_4^+-N and urea-N), which together accounted for the vast majority of total slurry N (see Methods). A small fraction of particulate organic N remained unlabeled and may have been partially taken up by plants without being traced in the fertilizer-derived N pool. As a result, estimates of fertilizer N recovery likely represent a conservative bound, with a minor (<5%) potential bias toward underestimating fertilizer-derived uptake and overestimating soil-derived N. However, this is unlikely to affect our central conclusion that plant N uptake was overwhelmingly dominated by soil-derived N across treatments. Second, biological N fixation was estimated using empirical relationships and assumed to be invariant among treatments, consistent with the absence of significant differences in nifH gene abundance under climate manipulation. Although this introduces uncertainty into ecosystem N budgets, BNF contributed only a minor fraction of total N inputs relative to slurry fertilization. Finally, because the experiment focused on slurry-based organic fertilization, observed responses may partly reflect interactions between climate change and slurry-derived carbon inputs. Future work should test whether similar patterns of climate-driven soil N mining occur under mineral fertilizer systems. Nevertheless, these uncertainties do not alter the main conclusion that climate change intensified plant reliance on soil-derived N despite intensive organic N fertilization" **(Lines 318–335)**.

2. The calculation of N balance is coarse. The authors estimated BNF based on previous empirical relationships derived from comparable grasslands. However, the studied montane grassland is intensive managed (with cattle slurry). This difference may bias the estimation of BNF.

Response: Thanks very much for this important comment. We agree that estimating biological N fixation (BNF) using empirical relationships derived from comparable grasslands introduces uncertainty, because the studied montane grassland is intensively managed with cattle slurry application. However, we consider this uncertainty unlikely to affect the central conclusions of the study for several reasons.

First, legumes constituted only a small fraction of total plant biomass (~3%), and estimated BNF contributed only a minor proportion of total ecosystem N inputs relative to slurry fertilization. Second, ecosystem N balance calculations were primarily used to provide supporting evidence for climate-driven soil N mining, which was independently and directly

supported by the observed increases in soil-derived plant N uptake and declines in soil total N pools (–3.0% to –17.8%). Third, nifH gene abundance did not differ significantly among treatments, suggesting that climate manipulation had limited effects on potential BNF activity.

Accordingly, although ecosystem N balances were estimated using currently available regional data and empirical relationships, they should be interpreted as indicative of relative treatment effects rather than precise ecosystem-scale N budgets. We therefore consider that this uncertainty does not alter the main conclusion that climate change intensified plant reliance on soil-derived N despite intensive organic N fertilization. We have now explicitly acknowledged and discussed these uncertainties in both the Methods and Discussion sections of the revised manuscript.

The added discussion about the uncertainty reads now "As the abundance of the nifH gene did not significantly differ among treatments (Fig. 3; Supplementary Fig. 5), BNF was assumed to be unaffected by climate manipulation. BNF was estimated using observed legume abundance (3% of total biomass) and established empirical relationships from comparable grasslands, yielding an average BNF rate of 15 kg N ha^{-1} yr^{-1}. We acknowledge that this approach may introduce uncertainty because the studied grassland was intensively managed with slurry application. However, legumes constituted only a small fraction of total biomass, and BNF contributed minimally to total ecosystem N inputs relative to slurry fertilization. Plant N export (Nharvest) was determined from measured shoot biomass and N concentration. Negative N balance values indicate net depletion of soil N pools. Although ecosystem N balances were estimated using currently available regional data and empirical relationships, the resulting ecosystem N balances should be interpreted as indicative of relative treatment effects rather than precise ecosystem-scale N budgets" (Methods section, **Lines 492–504**) and "Second, biological N fixation was estimated using empirical relationships and assumed to be invariant among treatments, consistent with the absence of significant differences in nifH gene abundance under climate manipulation. Although this introduces uncertainty into ecosystem N budgets, BNF contributed only a minor fraction of total N inputs relative to slurry fertilization" (Discussion section, **Lines 326–330**).

3. Cattle slurry addition introduces both C and N into the soil, thereby affecting both C and N availability. C availability may further influence microbial activity and its response to climate change. The current discussion does not mention the effect of C. The introduction also largely ignores the role of C addition. Therefore, the current results may reflect the specific conditions of organic fertilizer application rather than broader fertilization scenarios.

Response: Thanks very much for your insightful comment. We agree that cattle slurry application simultaneously introduces both C and N into soils, and that slurry-derived C may influence microbial activity and soil N transformations under climate change. We have now explicitly incorporated this aspect into both the Introduction and Discussion sections. In the

Introduction, we added the following statement: "In addition, slurry applications simultaneously introduce both carbon and nitrogen into soils, potentially stimulating microbial activity and altering soil N transformation under climate change" (**Lines 102–104**). In the Discussion, we further considered the potential role of slurry-derived C in promoting microbial decomposition and soil N mobilization: "In addition, eCO_2-induced increases in root exudation, combined with slurry-derived carbon inputs, likely further stimulated microbial decomposition of soil organic matter, intensifying microbial N mining" (**Lines 261–264**).

We also agree that the present results should be interpreted within the context of intensive organic fertilization systems. Accordingly, we have clarified this scope throughout the revised manuscript, including statements such as: "Collectively, these results demonstrate that climate change significantly strengthens plant reliance on soil-derived N, even under intensive organic N fertilization, in montane grassland ecosystems" (**Lines 273–275**), and "Nevertheless, these uncertainties do not alter the main conclusion that climate change intensified plant reliance on soil-derived N despite intensive organic N fertilization" (**Lines 334–335**).

At the same time, we discussed the broader relevance of our findings by citing recent evidence from chemically fertilized agroecosystems: "A recent study in rice-cropping systems similarly showed that chemical N fertilization can stimulate SON mineralization and promote soil-derived N losses and plant N uptake via the microbial N pump mechanism, suggesting that climate-driven soil N mining may represent a more general feature of intensively fertilized agroecosystems" (**Lines 296–300**).

Finally, we explicitly acknowledged this limitation and highlighted the need for future studies to evaluate whether similar responses occur under mineral fertilizer systems: "Because the experiment focused on slurry-based organic fertilization, observed responses may partly reflect interactions between climate change and slurry-derived carbon inputs. Future work should test whether similar patterns of climate-driven soil N mining occur under mineral fertilizer systems" (**Lines 330–333**).

Minor comments

4. Line 368, "0.2 g N kg-1 FW organic N" Does this organic N refer to unextractable organic N? Please specify.

Response: Yes. We have corrected the sentence to "…comprising 0.2 g N kg^{-1} FW unextractable organic N" (**Lines 407–408**).

5. Line 368-369, Please report the results of N forms and their individual contents (e.g., unextractable N, dissolved N (NH_4, urea-N, other dissolved N)). What is the proportion of urea in total dissolved N? I checked references [17] and [22] and did not find these results.

Response: Thanks for your comments. We have now reported the major N forms and their proportions in the cattle slurry in the Methods section: "On average, the slurry had a pH of 7.5 and a total nitrogen content of 2.0 g N kg^{-1} fresh weight (FW), comprising 0.2 g N kg^{-1} FW unextractable organic N, and 1.8 g N kg^{-1} FW dissolved N, including ammonium (NH_4^+-N) and urea-N" (**Lines 406–408)**.

We did not separately quantify the proportions of NH_4^+-N and urea-N within the dissolved N pool. Instead, the ^{15}N-labelling approach was designed to target the dominant plant-available N forms in fresh cattle slurry by adding equal N amounts of 99% ^{15}N-enriched ammonium sulfate and urea. Based on the dissolved N content of the slurry, this approach effectively labelled the majority (>85%, ~90%) of plant-available N forms. We acknowledge that the small fraction of unlabeled organic N introduces some uncertainty into estimates of fertilizer-derived N recovery and may slightly overestimate the contribution of soil-derived N. However, as discussed in our response to Comment 1, the estimated bias is minimal (<5%) and does not affect the central conclusion that plant N uptake was predominantly sustained by soil-derived N under climate change.

6. Line 368-369, Line 373, "label around 90% of all N forms in the slurry" A previous study reported "label around 3/4 of all N forms in the slurry." In addition, why did you cite reference [38] here?

Response: We apologize for the confusion. We have corrected this issue in the revised manuscript. As explained in our response to Comment 1, the ^{15}N-labelling efficiency in this study reflects the composition of the slurry used, which contained a higher proportion of dissolved plant-available N forms compared with the slurry described in the previously cited study. As a result, our system achieved a higher labelling efficiency (~85–90%) than that reported in the literature (~75%). We also agree that the citation of reference [38] in this context was inappropriate and has now been removed.

7. Line 434, labeling efficiency? Please clarify.

Response: We apologize for the confusion. We have clarified the wording in the revised manuscript by replacing "labeling efficiency" with "high labeling effectiveness for slurry N" to better reflect the description of the ^{15}N tracer application (**Line 474**).

We believe that these revisions have addressed the reviewers' concerns and improved the clarity and rigor of the manuscript. We thank the editor and reviewers for your constructive comments.

Yours sincerely,

Longlong Xia on behalf of all authors
